# α-Phenyl-*N-tert*-Butylnitrone and Analogous α-Aryl-*N*-alkylnitrones as Neuroprotective Antioxidant Agents for Stroke

**DOI:** 10.3390/antiox13040440

**Published:** 2024-04-07

**Authors:** José Marco-Contelles

**Affiliations:** 1 Laboratory of Medicinal Chemistry, Institute of Organic Chemistry (CSIC), C/ Juan de la Cierva, 3, 28006 Madrid, Spain; jlmarco@iqog.csic.es; 2 Centre for Biomedical Network Research on Rare Diseases (CIBERER), Center for Biomedical Network Research (CIBER), Carlos III Health Institute (ISCIII), 46010 Madrid, Spain

**Keywords:** antioxidants, neuroprotection, nitrones, reactive oxygen/nitrogen species, PBN, stroke

## Abstract

The recent advances in research on the use of the antioxidant and neuroprotective agent α-phenyl-*N-tert*-butylnitrone (PBN) for the therapy of stroke have been reviewed. The protective effect of PBN in the transient occlusion of the middle cerebral artery (MCAO) has been demonstrated, although there have been significant differences in the neuronal salvaging effect between PBN-treated and untreated animals, each set of data having quite large inter-experimental variation. In the transient forebrain ischemia model of gerbil, PBN reduces the mortality after ischemia and the neuronal damage in the hippocampal cornu ammonis 1 (CA1) area of the hippocumpus caused by ischemia. However, PBN fails to prevent postischemic CA1 damage in the rat. As for focal cerebral ischemia, PBN significantly reduces cerebral infarction and decreases neurological deficit after ischemia using a rat model of persistent MCAO in rats. Similarly, the antioxidant and neuroprotective capacity of a number of PBN-derived nitrones prepared in the author’s laboratory have also been summarized here, showing their high potential therapeutic power to treat stroke.

## 1. Introduction

Aging and neurodegeneration due to damage by free radicals (FRs) are very well-known biological events at the origin of stroke and neurodegenerative diseases (NDS) such as Alzheimer’s disease (AD), Parkinson’s disease (PD), and cancer, to mention some of the most devastating examples [1].

There is scientific evidence to support the relationship between stroke, or NDs, and the excessive production of reactive oxygen species (ROS) [2]. An accumulation of oxidative damage (OD) may contribute to the delayed onset and progressive nature of NDs [3]. One important factor contributing to neurodegeneration is that the central nervous system is unable to cope sufficiently with excessive FRs [2]. Thus, the neuroprotection strategy is considered one of the most attractive options to avoid FRs’ toxic effects [4]. Dementia due to cerebral ischemic lesions is relatively common in older people. In other words, multiple (micro)infarcts may cause dementia [5,6].

The ‘FR hypothesis of aging’ maintains that: (1) endogenous antioxidant defense mechanisms are insufficient to detoxify all oxygen FRs continually being generated, and (2) resulting OD to critical biological molecules, such as DNA, protein, and membrane lipids, contributes to age-related neuronal loss and/or dysfunction [7]. Supportive of this hypothesis are the studies showing increased OD during normal brain aging in AD and PD [8]. Consequently, ROS are important causative factors in normal brain aging and NDs, and chronic enhancement of antioxidant defenses should slow this process, resulting in improved cognitive and/or motor function.

On the other hand, excitotoxicity has been linked to oxidative stress (OS). *N*-methyl-D-aspartate (NMDA) has been shown to increase FR generation in vivo [9]. FR spin traps exert neuroprotective effects against both glutamate and NMDA toxicity in vitro [10]. It has been suggested that excitotoxicity and OS may be sequential and share interactive mechanisms, leading to neuronal degeneration [11,12].

Stroke is a condition affecting an increasing number of people worldwide and is the main cause of disability [13]. The ischemic cascade begins with energy failure due to the obstruction of a blood vessel that produces a massive and prolonged release of glutamate [14]. Physiopathological events associated with brain ischemia are related to OS process, Ca^2+^ dyshomeostasis, mitochondrial dysfunction, pro-inflammatory mediators, and/or programmed neuronal cell death [15]. In the ischemic stroke, as the result of the obstruction of a blood vessel, a critical reduction of oxygen–glucose supply and cerebral blood flow (less than 25%) occur in brain [16]. Thus, under deprivation of oxygen and glucose, cell death takes place in two phases: cell death from anoxia/hypoxia and energy depletion [17], followed by reperfusion that increases OS and FR formation, excitotoxicity, and nitric oxide (NO) production with ulterior energy failure and delayed death [18].

No effective therapeutic drugs to treat or prevent brain damage in ischemic stroke are available. Currently, the therapy for acute ischemic stroke has two basic principles: (1) dissolving the intravascular occlusion (***reperfusion***), which may refer to reperfusion injury (RI) (tissue damage caused when blood supply returns to the tissue) or reperfusion therapy (the medical treatment that restores blood flow through blocked arteries, typically after a ichemic attack), and (2) preserving the brain from the harmful cellular and metabolic cascade (***neuroprotection***). It is known that ischemia with RI leads to an enhanced production of FRs and that this production contributes to OD [19]. Even if ischemia is not followed by RI, significant amounts of ROS may be generated in brain tissue with some degree of residual perfusion, as demonstrated in the ischemic penumbra during occlusion of the middle cerebral artery (MCAO) in rats.

Nowadays, the only treatment approved for stroke is the recombinant tissue plasminogen activator (rtPA). rtPA is used to open a blood vessel, preventing brain damage at the ischemic penumbra, which, despite being a hypoperfused and non-functional tissue, is a viable tissue adjacent to the infarcted core. However, rtPA has a very narrow therapeutic window (3.5 h) [20].

Thus, in the search for alternative therapies to rtPa [21] to prevent its secondary effects and improve its narrow therapeutic window, new therapeutic agents are needed to recover tissue functionality before cell death and to be effective against several targets, including excitotoxicity and disturbed Ca^+2^ homeostasis [22], mitochondrial failure [23], OS and nitrosative stress [24], inflammation [25], and apoptosis [26].

**Figure 1:**
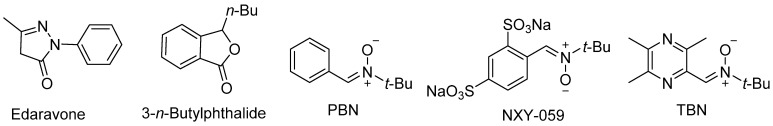
Structures of edaravone, *n*-butylphtalide, PBN, NXY-059 and TBN.

As part of this effort, two small molecules, edaravone [27] (Figure 1) and 3-*n*-butylphthalide [28] (Figure 1) have been recently approved for the therapy of stroke in Japan and China, respectively.

**Scheme 1:**
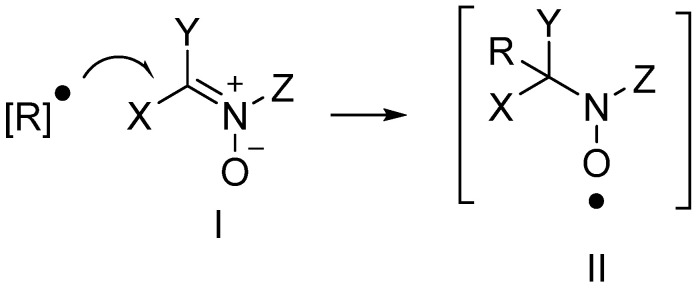
Reaction of species [R]^●^ with nitrone I leading to nitroxide II.

In addition, and in this context, nitrones [29,30] (I, Scheme 1) have been largely investigated as targets of choice. Nitrones are small spin-trapping organic compounds that readily react with a variety of FRs, forming unreactive, more stable spin-adducts, a property that was first used to detect FRs by electron paramagnetic resonance. There is significant evidence supporting the notion that FR production is a key factor in the development of brain injury following both cerebral ischemia (CI) and RI. The use of compounds which trap FRs to ameliorate ischemic damage is, therefore, a logical therapeutic approach. Thus, not surprisingly, nitrones occupy a privileged place for stroke and other human conditions, such as NDs [31], diseases of aging [32], cancer [33], cardiovascular disease [34], renal injury [35], visual loss [36], and acoustic trauma [37,38].

Nitrones are compounds that are able to trap ROS and reactive nitrogen species (RNS), such as ^●^OH, O_2_^●−^, NO^●^, and ONO_2_^−^, and non-radical molecules [hypochlorous acid, or hydrogen peroxide (H_2_O_2_)] [39], as common biological events involved in the progress of diseases linked to OS. Nitrones are potent antioxidant molecules [40] that are able to reduce OS. Nitrones’ power to scavenge the different types of ROS derives from their activated carbon–nitrogen double bond [R^1^R^2^C=N**^+^**(O**^−^**)R^3^ (I)] that prompts the easy reactive species [R]^●^ nucleophilic radical attack, leading to the less reactive and harmful nitroxide II species (Scheme 1) for biological targets, resulting in cell survival, tissue, biomolecules, and membrane stability [41]. However, recent results, such as the fact that the doses used for spin-trapping experiments are 1000-fold higher than those usually applied in the in vitro neuroprotection analyses (10–50 μM), and that the amounts of nitrones in vivo are currently under 50 μM (clearly insufficient to trap ROS/RNS), suggest that other mechanisms are responsible for nitrones’ scavenging capacity [39]. Nowadays, it is widely accepted that nitrones may suppress signal transduction processes, affording significant anti-inflammatory/anti-apoptotic [42] and NO-releasing properties [43] (these events being, most possibly, the origin of their antioxidant/neuroprotective profile [39]).

The literature is full of articles and reviews that have covered the organic [44], physicochemical/computational [45], and biological aspects of these molecules [46]. In addition, collateral topics, such as “antioxidants” [47], “ROS”, and “spin-trapping agents” [48], usually include nitrones in their accounts, resulting in an impressive mass of information underlying their medicinal chemical features [49].

Based on the understanding of the biochemical processes involved in the formation and development of a stroke, a number of products have been developed that target the different ischemic and RI events. Despite the promising initial results, neuroprotection drugs for stroke have failed in advanced clinical phases; consequently, no neuroprotective agent has been approved by the Food and Drug Administration (US) for stroke therapy.

However, neuroprotection is still a choice, and OS is a suitable biological target. In this context, antioxidants such as α-phenyl-*N-tert*-butylnitrone (PBN) (Figure 1) [50], NXY-059 (Figure 1) [51], and TBN [52] (Figure 1) have been developed, resulting in therapeutic candidates for cancer [53], NDs [54], hearing loss [42], and stroke [55].

NXY-059 (Figure 1) [56] is a well-known FR scavenger with a good neuroprotective profile in rat models of transient/permanent focal ischemia and in rodent models of stroke. It has been launched several times in advanced clinical studies without success [57]. In spite of this, *tert*-butylnitrones, such as NXY-059, are known to afford *tert*-butylhydroxylamines after hydrolysis as powerful radical scavengers that, further, could be oxidized to 2-methyl-2-nitrosopropane, which may then produce NO, the source and origin of the neuroprotection, as has already been reported for NO donor molecules [58].

On the other hand, recent reports have highlighted the powerful neuroprotective effect shown by new PBN derivatives bearing *N*-aryl substituents on human neuroblastoma cells under induced in vitro experimental OS [59]. In this study, we will focus on the neuroprotection power and capacities attributable to PBN and PBN-derived nitrones [39], the recent advances to prevent neuron death after stroke and RI [60]. This means, obviously, a selection of nitrones that we have restricted to PBN-analogues [39] (Figure 1), developed in the author’s laboratory. Other PBN-nitrones developed in other groups have already been reviewed [30,39] and are not going to be discussed here.

## 2. PBN, an Antioxidant and Neuroprotective Agent

PBN (Figure 1) is one of the best-known and investigated spin traps of ROS/RNS. PBN is actually a better scavenger for non-lipid radicals (such as hydroxyl, for instance) than for lipid radicals such as peroxyl and alkoxyl. PBN is both hydrophilic and lipophilic; it readily permeates all tissue, including brain tissue; its half-life in blood is 3–4 h [8]; and its concentration in plasma has been shown to peak at 15 min, while the maximum in other organs tested occurred at 30 min. When administered intraperitoneally (IP) to rats, the amount of PBN per gram of tissue is always higher in the liver and kidney than in the brain [61].

When injected into older adult gerbils, PBN led to the removal of oxidative damaged proteins, recovery of the ability to perform in a spatial radial maze test, reversal of age-related loss in the stimulation of dopamine release [62], affects on physiological functions, and extended life span in treated animals [63,64]. PBN releases NO after the reaction with ROS in vitro, and this release may play a key role in these activities.

Accumulating evidence has implicated ROS production and the resultant OD as a major contributing factor in brain aging and cognitive decline. It was shown [8] that chronic PBN treatment of 24-month-old rats for up to 9.5 months improved cognitive performance in several tasks, resulting in greater survival during the treatment period and decreased OD within brain areas important for cognitive function. These results not only provide a direct link between FRs/OD and cognitive performance in old age, but also suggest that antioxidants could be developed to treat or prevent age-related cognitive impairment and AD.

In 1990, in a preliminary communication [65], and in 1991, in a full paper [66], Phillis and Clough-Helfman reported that the IP administration of PBN (100 mg/kg) 30 min prior to transient MCAO bilateral 5 min (forebrain ischemia) in gerbils prevented the increase in locomotor activity observed in saline-injected ischemic animals and significantly reduced OD to the hippocampal cornu ammonis 1 (CA1) pyramidal cell layer observed 5 d post-ischemia. Measurements of body temperature revealed that the administration of PBN and the induction of CI were associated with small reductions in body temperature, but these changes were not significant. Finally, it was observed that PBN (100 mg/kg) administered 2 h post-ischemia failed to protect against CI. Overall, these findings support the hypothesis of an involvement of ROS as a significant cause of ischemia-RI-induced cerebral injury, and suggest that PBN may be a useful agent for the prevention of CI—the protective effect in ischemia/RI being related to its ability to prevent a cascade of FR generation by forming spin-adducts.

In 1993, Sen and Phillis [67] communicated that a combination of systemic and topical PBN (100 mM) was required to suppress hydroxyl radical formation during CI/RI, as PBN FR adducts were detected in EPR spectra of the lipid extracts of PBN-treated rat brain subjected to CI/RI.

In 1992, Yue and co-workers [68] described the ability of PBN to attenuate ischemia-induced forebrain (global brain ischemia) edema and hippocampal CAl neuronal loss in gerbils and to protect rat cerebellar neurons in primary culture from glutamate-induced toxicity. Thus, PBN, given IP at 75 or 150 mg/kg 30 min before CI (5 min MCAO) increased survival (7 d) of CAl neurons from 60 ± 14 (vehicle treated, n = 17) to 95 ± 15 (*p* < 0.05, n = 15) and 145 ± 3 (*p* < 0.01, n = 15), respectively. When gerbils were treated with PBN (50 mg/kg, IP) immediately and 6 h after RI, followed by the same dose for an additional 2 d, CAl neurons survival improved from 35 ± 9 (vehicle, n = 20, 6 min MCAO) to 106 ± 17 (*p* < 0.01, n = 13). In gerbils exposed to a more severe CI (10 min), pre-treatment with 150 mg/kg PBN increased the survival of CAl neurons from 6 ± 6 (vehicle) to 27 ± 10 (*p* < 0.05, n = 11). Pretreatment with PBN, at 150 mg/kg, reduced forebrain edema (following 15 min CI) by 24.7% (*p* < 0.01, n = 16). Note that PBN at 50 mg/kg, IP, had no hypothermic effect and at 75 or 150 mg/kg caused a transient hypothermia. The presence of PBN in the brain was confirmed in microdialysis samples and brain tissue extracts using HPLC. Finally, it was reported that in vitro, PBN protected rat cerebellar neurons against 100 µM glutamate-induced toxicity (EC_50_ = 2.7 mM). These results further support the concept that FRs contribute to brain injury following CI and suggest the potential therapeutic application of electron spin traps in stroke.

In 1994, Zhao et al. [69] observed that, in a transient MCAO experiment in rats, PBN dramatically reduced infarct size when given 1 or 3 h after recirculation following a 2 h period of MCAO. PBN was dissolved in saline (10 mg mL^−1^ or 20 mg mL^−1^) and administered intraperitoneally. Four groups of animals were studied: one injected with saline (n = 16), and three with PBN (n = 8, n = 10, and n = 11). Four doses of PBN 100 mg kg^−1^ were given, with an interval of 12 h. The initial dose of PBN was either given 15 min before MCAO or 1 h or 3 h after recirculation. Eight control animals were given saline 15 min before CI and another eight animals were given saline post-ischaemically in four doses. Since the results were similar, the two control groups were pooled. Blood pressure, PO_2_, PCO_2_, pH, and blood glucose were measured before and after the induction of CI and at the end of the 2 h ischaemic period. Temperature was controlled during CI and for the first 4 h of recirculation. These results, and those reported by Cao and Phillis (see below) [46], demonstrate that the effect of PBN in ameliorating infarct size following MCAO surpasses that of any other drug tested before, as no other drug reduced the infarct size to 50% of control or less when given as late as 5 h after permanent MCAO or 3 h after recirculation, following 2 h MCAO.

In 1994, Cao and Phillis [46] reported the first study to demonstrate protection by PBN treatment against permanent focal MCAO and ipsilateral common carotid artery occlusion in rats. Studies on the metabolism and distribution of PBN in rats have shown that PBN is rapidly absorbed after IP injection in rats. The PBN level in plasma peaks at 15 min and decreases steadily over the subsequent 12 h, with a half-life in blood plasma of 2–3 h. The concentration of PBN in the brain peaks 30–45 min after administration and then decreases steadily during the next 8 h. The brain concentration of PBN is significantly higher than that in blood. The increased brain distribution of PBN was attributed to its greater lipophilicity and its ability to penetrate the blood–brain barrier (BBB). PBN was selected as the spin-trapping agent and its times of administration were scheduled based on the above results. The doses of PBN used in the present study replicated those used in previous studies by Phillis and Clough-Helfman [65,66]. Thus, PBN was given IP at 100 mg/kg at initial times of 0.5 h prior to CI (group 2) and 0.5 (group 3), 5 (group 4), and 12 h (group 5) after CI. Additional doses of PBN (100 mg/kg) were administered as follows: group 2 at 24 h; group 3 at 5, 17, 29, and 41 h; group 4 at 17, 29, and 41 h; group 5 at 24 and 36 h. Animals were sacrificed 48 h after MCAO and infarct volumes were calculated from 2,3,5-triphenyltetrazolium chloride (TTC)-stained 1.5 mm slices of the forebrain. PBN significantly attenuated cortical infarct volume and cerebral edema in all of the treated rats compared with those in ischemic control (group 1), with no significant differences between the different PBN treated groups. The percentage of infarct volume in ischemic control rats was 22.7 ± 1.0, while those in PBN-treated groups were 9.6 ± 2.0, *p* < 0.01 (group 2); 12.2 ± 2.2, *p* < 0.01 (group 3); 11.1 ± 2.9, *p* < 0.01 (group 4); and 14.4 ± 2.5, *p* < 0.01 (group 5). Thus, these results indicate that PBN (100 mg/kg) treatment, initiated both prior to and for up to 12 h after the onset of CI, significantly reduced cortical infarct volume and brain edema at 48 h post-ischemia. Furthermore, neurological behavior tests showed that PBN decreased the neurological deficit scores (NDS) in rats initially treated either prior to or for up to 12 h after CI. There was an indication that if treatment was initiated later, following MCAO, there would be a reduction in the protective effect. Although pretreatment with PBN may appear to be therapeutically impractical, since most acute strokes occur without warning, PBN administration also had a significant neuroprotective effect when its administration was initiated 12 h after CI.

To sum up, whereas the therapeutic time window for NMDA antagonists, non-NMDA antagonists, and glutamate release inhibitors in focal models of CI appears to be about 1–2 h, PBN still showed neuroprotective efficacy even when it was administered 12 h following focal CI. These findings demonstrate that PBN has a strong neuroprotective effect, supporting the hypothesis that FRs play an important role in brain injury following CI, and suggest that PBN could have potential therapeutic value for the treatment of stroke [46].

In 1995, Siesjö and colleagues [70] communicated the results of a study targeted to explore whether PBN influences the recovery of energy metabolism in rats following transient focal CI. MCAO of 2 h duration was induced in rats by an intraluminal filament technique and brains were frozen in situ at the end of CI and after 1, 2, and 4 h of recirculation. PBN was given 1 h after recirculation. Neocortical focal and penumbra areas were sampled for analyses of phosphocreatine (PCr), creatine, ATP, ADP, AMP, glycogen, glucose, and lactate. The penumbra showed a moderate-to-marked decrease and the focus showed a marked decrease in PCr and ATP concentrations, a decline in the sum of adenine nucleotides, near-depletion of glycogen, and an increase in lactate concentration after 2 h of CI. Recirculation for 1 h led to only a partial recovery of energy state, with little further improvement after 2 h and signs of secondary deterioration after 4 h, particularly in the focus. After 4 h of recirculation, PBN-treated animals showed significant recovery of energy state, with ATP and lactate contents in both focus and penumbra approaching normal values.

To sum up, it was found that PBN allowed recovery of ATP and lactate contents in tissues with dense CI and low ATP values during a 2 h period. In theory, this could reflect the accumulation of PBN in mitochondria, where it could conceivably prevent FR damage to components of the respiratory chain—PBN improving the microvascular function. Thus, these results unequivocally demonstrate that PBN markedly improves the recovery of cerebral energy state and mitochondrial metabolism [70].

In 1996, Pahlmark and Siesjö [71] described the results of the administration of PBN in a transient forebrain CI experiment in rats, either 30 min before or 30 min or 6 h after CI, with PBN or its vehicle. Thus, after 15 min of two-vessel MCAO in anaesthetized rats, brain damage was assessed by histopathological techniques 7 d later. PBN reduced neuronal necrosis in the neo-cortex when given 30 min post-treatment but not when given before or 6 h after CI; it also failed to reduce damage to the hipocampal CA1 or the caudoputamen. This result was totally unexpected because, although PBN has little effect on neuronal damage due to forebrain CI, it has been reported that PBN dramatically reduces infarct size due to permanent or transient MCAO in the same species [46,69]. These results and are in disagreement with those obtained in gerbils subjected to a similar type of forebrain ischemia [72,73]. Thus, it is tentatively concluded that while PBN ameliorates either the microvascular dysfunction or the mitochondrial failure, which could be the crucial events leading to infarction in focal CI, it has only a weak effect on the mechanisms that yield selective neuronal necrosis in transient brief CI.

In 1997, Jenkins et al. [74] communicated the effects of PBN on focal CI/RI in halothane-anesthetized rats after 90 min MCAO and RI. Intravenous injections of 25 mg/kg PBN 5 min before and 30 min after the insertion of a filament significantly attenuated the lesion when the volume was measured 24 h after CI. During CI and during the first 30 min after RI, cerebral blood volume and blood flow were measured by volume-sensitive and newly developed flow-sensitive magnetic resonance imaging techniques and by laser– Doppler flowmetry. In all the animals, the area of decreased blood flow was larger than the area of decreased volume by a factor of 2.2. The area of the postrefusion flow deficits matched the final lesion volumes at 24 h measured histologically much better than did the blood volume deficits on both saline and PBN-treated animals. RI led to a return of blood flow and volume to values close to the contralateral side in the PBN-treated animals, in contrast to the saline-treated control group. It was concluded that in focal CI/RI, PBN provides protection of vascular endothelium, leading to enhanced postischemic RI, a result that suggests that the vascular endothelium may be a primary target for the damaging action of FRs given the protection afforded by PBN.

In 1997, Grotta and co-workers [75] showed that PBN reduced the damage produced by global and focal CI, with the latter being reduced even if PBN was administered 5 and 12 h after MCAO in reversible and permanent models in rats, respectively. Similar to previous reports [46,68,69], Grotta et al. demonstrated that PBN administered before CI dramatically reduces RI, resulting in an infarct volume indistinguishable from that produced by permanent CI. However, in contrast to PBN pretreatment and reports on PBN neuroprotection after delayed post-treatment [46,69], administration of PBN 2 h after MCAO (1 h before RI) was ineffective in reducing RI in the used ischemial reperfusion model. This lack of post-treatment efficacy casts doubts on the central importance of FRs in causing RI in this model [46] and suggests that PBN pre-treatment has a positive effect on RI by some other mechanism(s) for PBN neuroprotection. Because of the reported [46] unusually long (12 h) window for protection with PBN using a permanent focal CI model with no RI, it is possible that, to provide ultimate neuroprotection, PBN must interact with a type of cellular processing that occurs during CI rather than during RI. For instance, it has been described that irreversible loss of Ca^2+^/calmodulin-dependent protein kinase II (CaM-KIT) activity, occurring during early CI, correlated with neuronal damage after global and focal CI [76] and that NMDA antagonists and hypothermia, which were able to decrease CaM-KII inactivation, were effective in decreasing brain damage [77]. Interestingly, PBN has been shown to completely inhibit inactivation of CaM-KII after CI in gerbils [78], suggesting that prevention of CaM-KIl inactivation or improvement of its recovery is one possible way that PBN may protect the brain from CI.

In 1999, Green and co-workers [79] described their attempts to determine whether delayed treatment with PBN was protective at 2 months following transient global forebrain CI and whether additive effects can be observed when PBN is administered in combination with moderate hypothermia. For this objective, rats were subjected to 10 min of two-vessel forebrain CI followed by 3 h of postischemic normothermia (37 °C); 3 h of postischemic hypothermia (30 °C); normothermic procedures, combined with delayed injections of PBN (100 mg/kg) on days 3, 5, and 7 post-insult; and postischemic hypothermia combined with delayed PBN treatment, or sham procedures. Cognitive behavioral testing and quantitative histopathological analysis at 2 months were carried out. It was observed that postischemic PBN injections induced a systemic hypothermia (1.5–2.0 °C) that lasted for 2–2.5 h. Water maze testing revealed significant performance deficits relative to shams in the normothermic ischemic group, with the postischemic hypothermia and PBN groups showing intermediate values. A significant attenuation of cognitive deficits was observed in the animal group receiving the combination postischemic hypothermia and delayed PBN treatment. Quantitative hippocampal CA1 cell counts indicated that each of the ischemia groups exhibited significantly fewer viable CA1 neurons compared to sham controls. However, in rats receiving either delayed PBN treatment or 3 h of postischemic hypothermia, significant sparing of CA1 neurons relative to the normothermic ischemia group was observed.

To sum up, these data indicate that hypothermia combined with PBN treatment provides long-term cognitive improvement compared to non-treated groups. Thus, PBN-induced mild hypothermia could contribute to the observed neuroprotective effects [79].

In 1999, Niwa et al. [80] compared the effects of PBN and edaravone (Figure 1), evaluating them in a rat transient MCAO model to clarify the possible role of ROS in the RI. The volume of cerebral infarction, induced by 2 h MCAO and subsequent 2 h RI in Fisher-344 rats, was evaluated once PBN (100 mg/kg) and edaravone (100 mg/kg), administered just before reperfusion of MCAO, had been significantly reduced by a similar percentage. Edaravone significantly prevented ^•^OH-induced hydroxylation of salicylate but did not influence superoxide generation; it significantly reduced the infarction volume following transient focal brain CI by a similar percentage to PBN. In these preliminary results, the same dose of edaravone significantly diminished the brain edema caused by transient CI in this model. From these observations, it was concluded that ^•^OH is mainly responsible for the cerebral RI after transient focal CI.

In 2000, Zausinger et al. [49] reported that treatment with PBN significantly reduced cortical infarction (−31%) in a 90 min reversible unilateral MCAO in rats. The same team [81] reported the observed effects in rats subjected to 90 min of MCAO in a rat model of reversible focal CI, local cerebral blood flow (LCBF) bilaterally recorded by laser Doppler flowmetry, NDSs quantified daily, and infarct volume assessed after 7 d. Thus, MCAO reduced ipsilateral LCBF to 20–30% of baseline. After RI, postischemic hyperemia was followed by a decrease in LCBF to about 70% of baseline; the improvement of neurological function and reduction of infarct volume (25%) in animals treated with PBN was not significant.

**Figure 2:**
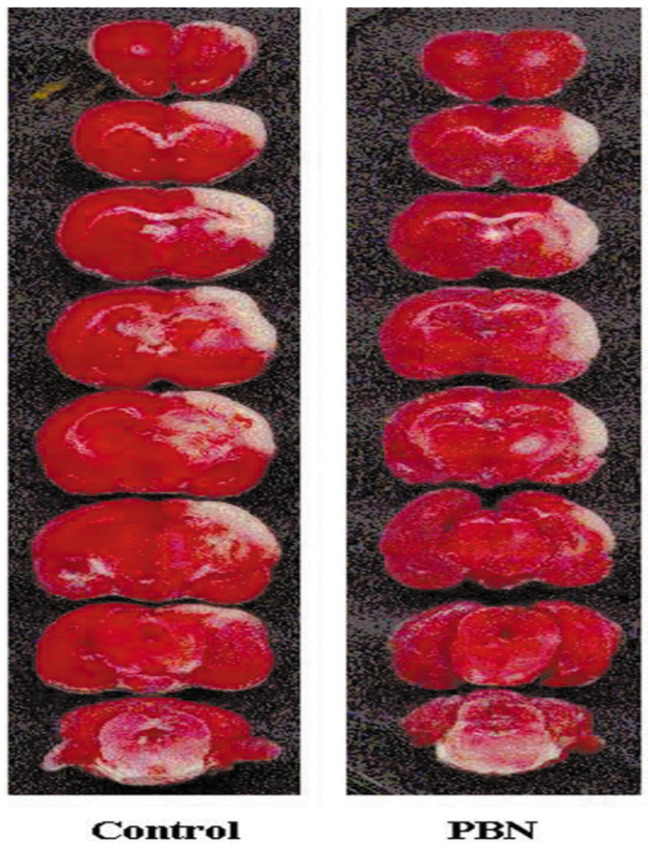
Representative TTC-stained images of brain coronal sections from animals in different groups. Embolization of the MCAO with the thrombus resulted in extensive brain infarction in the affected hemisphere. Compared to placebo-treated animals, there was significantly better protection in the animals treated with PBN at 2 h after the ischemic insult. Reproduced with permission from ref. [82]. Copyright 2000. Elsevier.

In 2000, Yang and colleagues [82] evaluated the neuroprotective effect of PBN compared to a vehicle in a focal embolic MCAO in rats. Wistar rats were randomly divided into three groups (n = 10, each group). Animals in the control group received the vehicle and those in the treatment groups were treated with PBN, (both 100 mg/kg/d × 3 d, IP) starting 2 h after the introduction of an autologous thrombus into the right-side MCAO. The neurological outcome was observed and compared before and after treatment and between groups. The percentage of infarct volume was estimated from TTC stained coronal slices 72 h after the ischemic insult. 2 h post–ischemia administration of PBN significantly improved NDS at 24 h following MCAO embolization (both *p* < 0.01). The percentage of infarct volume for animals receiving the vehicle was 32.8 ± 9.4%. 2 h-delayed administration of PBN achieved a 35.4% reduction in infarct volume in treatment groups when compared with animals receiving the vehicle (PBN vs. control, 21.2 ± 10.9% vs. 32.8 ± 9.4%; *p* < 0.05) (Figure 2). These data indicate that free radical generation may be involved in brain damage in this model and that 2 h delayed postischemia treatment with PBN may have neuroprotective effects in focal CI.

Furthermore, there was no significant difference in the attenuation of cerebral ischemic lesion between various groups with administration of PBN with an initial time of 0.5 h prior to CI and 0.5, 5, and 12 h after CI [82].

Li and co-workers [83], based on the potent neuroprotective effect of PBN when administered after transient focal CI, and to further elucidate the mechanism of PBN action, have studied its effect on animal survival, histopathological outcome, and activation of caspase-3 following 30 min of global CI in vehicle- and PBN-treated rats. The results showed that 30 min of global ischemia was such a severe insult that no animal could survive beyond 2 d of RI. Histopathological evaluation showed severe tissue edema and microinfarct foci in the neo-cortex and thalamus. Close to 100% damage was observed in the *stratum* and hippocampal CA1 and dentate gyrus subregions. Post-ischemic PBN treatment significantly enhanced animal survival and reduced damage in the neo-cortex, thalamus, and hippocampus. Immunohistochemistry demonstrated that caspase-3 was activated following ischemia in the striatum and the neo-cortex. PBN suppressed the activation of caspase-3 in both structures. It was concluded that PBN is a potent neuroprotectant against both local and global CI. Besides its function as a FR scavenger, PBN may reduce ischemic brain damage by blocking cell death pathways that involve caspase-3 activation.

Despite the fact that PBN is a well-established and useful neuroprotective agent in CI in vivo models, to date, there is little information concerning its effectiveness when administered in combination with rtPa.

In 2001, Lapchak et al. [84] determined the effects of PBN when administered before rtPA on hemorrhage and infarct rate and volume. A total of 165 male New Zealand white rabbits were embolized by injecting a blood clot into the MCA via a catheter. 5 min after embolization, PBN (100 mg/kg) was infused intravenously. Control rabbits received saline—the vehicle required to solubilize the spin traps. In rtPA studies, rabbits were given intravenous rtPA starting 60 min after embolization. Post-mortem analysis included the assessment of hemorrhage, infarct size and location, and clot lysis. In the control group, the hemorrhage rate after a thromboembolic stroke was 24%. The amount of hemorrhage was significantly increased to 77% if the thrombolytic rtPA was administered. In the combination drug–treated groups, the PBN/rtPA group had a 44% incidence of hemorrhage. rtPA and PBN/rtPA were similarly effective at lysing clots (49% and 44%, respectively) compared with the 5% rate of lysis in the control group. There was no significant effect of drug combinations on the rate or volume of infarcts. This study suggests that PBN may have deleterious effects when administered after an embolic stroke. However, PBN, when administered in combination with rtPA, may improve the safety of rtPA by reducing the incidence of rtPA-induced hemorrhage.

In 2003, Christensen and co-workers [85] analyzed rats in permanent MCAO and treated 1 h after occlusion with a single dose of PBN (100 mg/kg) or saline. Body temperature was measured and controlled for the first 24 h to obtain identical temperature curves in the two groups. Cortical infarct volumes were determined on histological sections 7 d later. PBN did not significantly reduce infarct volume (control 28.3 ± 16.3 mm^3^ vs. α-PBN 23.7 ± 7.4 mm^3^). PBN, administered shortly following the induction of CI on infarct volume after an extended survival period of 1 week in the temperature-controlled rat model of permanent MCAO, did not reduce infarct size.

Although it has been suggested that FRs are involved in the genesis of ischemic brain damage, as shown by the protective effects of PBN in ischemic cerebral injury, Goda et al. have investigated the involvement of FRs in transient ischemic-induced delayed neuronal death [86]. However, the protective effect of PBN on the cerebral damage caused by MCAO–reperfusion in rats is known [69]. PBN has also been reported to reduce cortical infarct and edema in rats subjected to focal CI [46]. When administered either 30 min prior to or 30 min post-CI, PBN significantly reduced the degree of neuronal damage and loss in the hippocampal CA1 region [66]. These findings indicated that FRs contribute to necrotic, but not apoptotic, neuronal damage. However, Goda’s results indicate that PBN does not suppress delayed neuronal death and that FRs do not appear to contribute to hippocampal CA1 injury following transient forebrain CI [86].

## 3. Neuroprotection of PBN-Derived Nitrones from Author’s Laboratory

In looking for new potent PBN analogues with improved spin-trapping power and strong neuroprotection, in the last ten years I have designed, synthesized, and evaluated a number of simple, easily available PBN-derived nitrones whose neuroprotective and antioxidant profile will be highlighted in this section [87,88,89,90,91,92,93,94,95,96].

### 3.1. Neuroprotection of PBN-Derived Nitrones 1–13

**Figure 3:**
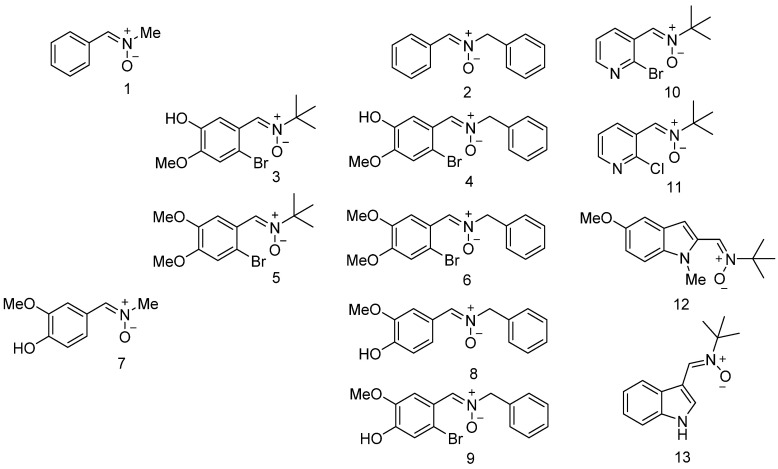
Structure of PBN-derived nitrones 1–13.

In the 2011 *BMC* paper [87], we reported the synthesis, structure, theoretical, and experimental in vitro antioxidant properties, using the DPPH and ORAC methods, as well as the neuroprotective activities, of PBN-derived nitrones 1–13 (Figure 3), with nitrone PBN being used as an internal reference compound. The synthesis of these nitrones was carried out as usual [87] from the corresponding commercially or easily available carbaldehydes, which were isolated and characterized as pure and single *Z*-isomers by the current analytical and spectroscopic methods [87].

The antioxidant activity of PBN-derived nitrones 1–13 (Table 1) was evaluated by the DPPH and ORAC assays. Regarding the antioxidant activity, for the DPPH radical test, as expected, nitrones 7 and 8 bearing free phenol groups gave the best RSA (%) values (87.6% and 88.2%, respectively). Note also that, out of the five nitrones bearing a phenol group, the most potent in the DPPH test was nitrone 8, bearing a hydroxyl group and no bromine atom in *para* and *ortho* positions, respectively, with respect to the nitrone moiety. In the ORAC analysis, the most potent radical scavenger was nitrone indole 12 (7.98 μM Trolox/μM compound), followed by the *N*-benzyl benzene-type nitrones 4 (7.36 μM Trolox/μM compound) and 9 (6.40 μM Trolox/μM compound). Thus, the most potent in the ORAC assay was nitrone 4, bearing a hydroxyl group and a bromine atom in *meta* and *ortho* positions, respectively, with respect to the nitrone group. Very interestingly, PBN gave a low RSA (%) value of 1.4% in the DPPH test and was inactive in the ORAC assay. Concerning its ability to scavenge ^•^OH, since this species is able to react with everything, limited only by its diffusion speed, it was not surprising that this scavenging assay could not differentiate the compounds. Consequently, the nitrones studied proved active in this experiment, showing high values in the 90% range—the most potent being nitrone 8 (96.9%). The neuroprotection of the of PBN-derived nitrones 1–13 (Figure 3) was assayed in human neuroblastoma cells stressed with a mixture of rotenone/oligomycin-A. Thus, we found that it was in general very low, with values ranging from 0% to 18.7%, the most potent being nitrones 1, 2, and 10, showing values in the range 24–18.4%. Overall, the nitrones studied in the 2011 BMC paper [87] showed a good antioxidant power according to the DPPH and ORAC tests but modest neuroprotective profile against the rotenone/oligomycin-A insult. Furthermore, there were no parallel activity power between the most potent antioxidant and neuroprotective nitrones; consequently, no clear structure–activity relationships could be established.

Later, in our 2012 *JMC* paper [88], additional antioxidant studies on different tests, such as the in vitro inhibition of soybean lipoxygenase (LOX), the inhibition of lipid peroxidation (LP) induced by AAPH, the competition of the tested compounds with DMSO for hydroxyl radicals, the superoxide anion radical (O_2_^•−^) scavenging activity, and the NO donating power, were carried out on the same PBN-nitrones 1–13 using PBN as the standard nitrone. As shown in Table 2, the most potent LOX inhibitors are the *N-tert*-butyl nitrones 10 (88%) and PBN (83%), presenting higher activity than the reference compound NDGA (40%). Thus, it seems that the inhibitory effect can mainly be attributed to the presence of the *N-tert*-butyl moiety 11–13, as the replacement of *N-tert*-butyl by *N*-methyl or *N*-benzyl groups diminishes/suppresses inhibition (e.g., 1 with *N*-methyl and 2 with *N*-benzyl group). Nitrones 1, 6, 7, 9, 11–13 exhibit weaker inhibition. Nitrones 2–5 and 8 do not present any biological activity under the reported experimental conditions. Furthermore, it seems that within the pyridinyl analogues, bulkier and more lipophilic substituents lead to more potent LOX inhibitors, as the 2-Br derivative 10 (π-Br = 0.86, MR- Br = 0.888) exhibits higher activity than the 2-Cl substituted analogue 11 (π Cl = 0.71, MR-Cl = 0.63). Compounds incorporating phenol moieties do not seem to be particularly effective agents. Although lipophilicity is referred to as an important physicochemical property for LOX inhibitors, among the tested nitrones the most potent compound **10** has a % inhibition value of 88% and clog *P* 2.01, which does not follow this trend. On the contrary, nitrone 6, with the higher lipophilicity value, shows low anti-LOX activity (21%, Table 1). In general, the inhibitory activity of the nitrones does not seem to be correlated with their antioxidant activity. Since these molecules do not bear a close and chemical similarity to the arachidonic acid (the substrate of LOX), it is possible that they might bound not to the active site but to a separate enzyme site. As shown in Table 2, with the exception of compounds PBN and 6, which did not show any inhibition, all other compounds caused inhibition of LP. Nitrones 1, 5, 10, 11, and 13 showed inhibition values (5–57%) lower than the common standard Trolox (63%). Obviously, the absence of hydrogen atoms (phenolic hydroxyl groups) has a direct impact on these results. On the other hand, nitrones 2–4, 7–9, and 12 gave higher values (67–90%) than Trolox, the most potent inhibitors being the nitrones 7 (90%) and 8 (90%). Nitrones 3, 4, 7, 8, and 9 might exert their antioxidative effect through their easily oxidizable hydroxyl unit. The antioxidative effect of nitrone 12 should be attributed to its interception of the alkylperoxyl radicals, mainly by the indole moiety, and possible formation of oxidized derivatives. Lipophilicity, as clog*P* theoretically calculated values using the CLOG *P* program, does not seem to influence their interaction. As shown in Table 2, with the exception of nitrones 4, 6, and 7, which did not show any result at 0.1 mM concentration, and nitrone 11, showing 30% scavenging activity, all the other compounds significantly compete with DMSO at 0.1 mM, giving, in some cases, values higher than Trolox (73%) for scavenging the hydroxyl radical (HO^•^). The most potent nitrones were 2 (97%), 10 (93%), and PBN (90%). It seems that lipophilicity does not play any significant role. As shown in Table 2, most of the nitrones showed poor or no capacity for scavenging activity of the superoxide anion radical (O_2_^•^). Only nitrone 2 presented a modest 24% scavenging activity. Finally, very low (if any) formation of NO_2_^−^ was observed from nitrones 7 (7%) and 8 (9%) (Table 2). To sum up, from these results, we conclude that, among the new compounds investigated in the 2011 BMC paper [87], in decreasing order, nitrones 2, showing the capacity to inhibit LP (74%), trap HO^•^ (97%), and O_2_^•^(24%), and 8, showing the capacity to inhibit LP (90%), trap ^•^OH (52%), and NO (9%), were the most balanced, taking into account the different tests assayed, standard nitrone PBN being the most powerful [LOX (83%), HO^•^ (90%), O_2_^•^ (15%)] [88]. In the 2012 *JMC* [88] paper, selected compounds from Table 2, previously investigated in the 2011 *BMC* paper [87], such as nitrones 1, 4, 7, 8, and PBN showed no neuroprotective activity in the in vitro oxygen-glucose deprivation (OGD) model.

### 3.2. Neuroprotection of PBN-Derived Nitrones 14–20

**Figure 4:**
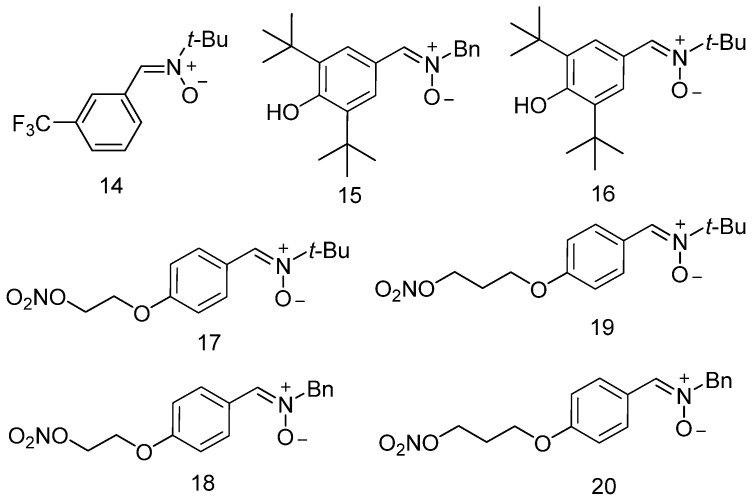
Structure of the PBN-nitrones 14–20 [88].

In our 2012 *JMC* [88] paper, we reported the synthesis and theoretical calculations; the antioxidant, anti-inflammatory, and neuroprotective properties; and the ability to cross the BBB of the PBN-derived nitrones 14–20 (Figure 4) as potential agents for stroke treatment. Target PBN-derived nitrones 14, 15, and 16 and the new PBN-derived nitrones 17–20 (Figure 4) were synthesized by reacting the corresponding commercial 3-(trifluoromethyl)benzaldehyde and 3,5-di-*tert*-butyl-4-hydroxybenzaldehyde, or from the readily available 3-(4-formylphenoxy)propyl nitrate and 2-(4-formylphenoxy)ethyl nitrate, with *N*-*tert*-butyl- or *N*-benzylhydroxylamine. Note that nitrones 17–20, bearing linkers, based on two or three methylene units between the nitrate group and the oxygen, attached to the aromatic ring, were designed as NO-donors, thus exploiting the potential pharmacological power associated with nitrate esters.

Regarding the in vitro inhibition of LOX, as shown in Table 3, among the PBN-nitrones tested, the most potent representative was compound **14** (65%) followed by 19 (57%), whereas for nitrones 15 and 16 no inhibitory activity was observed, and nitrones 17, 20, and 18 showed moderate inhibition. As shown in Table 3, nitrone 15 is a very interesting inhibitor of LP induced by AAPH (61%) and close to that of Trolox (63%), used as a reference compound. Compounds **14** and **17** did not show any inhibition value. The remaining nitrones exhibited significant inhibition of LP, the most potent being nitrone 20 (81%). Regarding the competition of the tested compounds with DMSO for hydroxyl radicals, as shown in Table 3, nitrones 17, 20, and 18 exhibited 98–100% scavenging activity, whereas 15 (77%), 14 (60%), and 19 (54%) seemed to follow. Nitrone 5 did not present any result under the experimental conditions. For the superoxide anion radical (O_2_^•^) scavenging activity, as shown in Table 3, only nitrone 14 showed a low but significant 29% scavenging activity. Finally, regarding the NO donating power, using the Griess reaction, virtually no formation of NO_2_^−^ was detectable in the absence of *L*-cysteine for 14, 15, 16, and 17–20, the present study demonstrating that, under the reported experimental conditions, the NO release from the tested compounds is very limited and low and seems to be independent of time [88]. From the observed results of the antioxidant capacity of nitrones 14, 15, 16, and 17–20 [88], we conclude that (*Z*)-2-methyl-*N*-(3-(trifluoromethyl)benzylidene)propan-2-amine oxide (14) is a potent inhibitor of LOX (65%) and efficiently scavenges HO^•^ (60%) and O_2_^•^ (29%), followed by (*Z*)-*N*-(4-(3-(nitrooxy)propoxy)benzylidene)-1-phenylmethanamine oxide (20) that inhibits LP (81%) and efficiently scavenges HO^•^ (98%), whereas (*Z*)-2-methyl-*N*-(4-(3-(nitrooxy)propoxy)benzylidene)propan-2-amine oxide (19) is a very well-balanced antioxidant agent as it inhibits LOX (57%) and LP (49%) and efficiently scavenges HO^•^ (54%) and O_2_^•^ (2%) [88].

First of all, the neuroprotective effect of PBN-nitrones 14–20 (Figure 3) was evaluated on primary neuronal cultures from the cerebral cortex, cultured for 6 to 8 d and subjected to OGD, under the usual experimental conditions. Not surprisingly, only nitrones 14 and 20 showed potent neuroprotection. Thus, the addition of 100–250 μM nitrone 14 and of 10–100 μM nitrone 20 significantly increased cell viability during reperfusion and returned near control values (100.3–95.7%, 93.4%, 94.4–101.2%, and 94.9–96.1%, respectively, compared to 100% control). The neuroprotection induced by nitrones 14 and 20 was compared with citicoline and PBN, these nitrones providing significantly higher neuroprotection than citicoline (Table 4).

Next, and in order to confirm the neuroprotective effects of PBN-nitrones 14–20 on cell viability, the effect of these compounds on necrotic cellular death was measured by monitoring LDH release from the cultured neuronal cells. As shown in Table 5, maximal neuroprotective effects (statistically significant when higher than 50% neuroprotection) were obtained at 250 μM nitrone 14, an effect comparable with citicoline or PBN at 100–250 μM and a smaller efficiency (near to 50% neuroprotection) at 100–250 μM nitrone 15, 100 μM nitrone 19, 250 µM nitrone 17, and 1–10 μM nitrones 20 and 18.

To sum up, from the 2012 *JMC* paper [88], we conclude that the majority of the PBN-derived nitrones 14–20 (Figure 4) compete with DMSO for hydroxyl radicals and that most of them are potent LOX inhibitors. Cell viability-related (MTT assay) studies clearly showed that nitrones 14 and 20 give rise to significant neuroprotection. When these nitrones were tested for necrotic cell death (LDH release test), PBN-nitrones 1 and 9 proved to be neuroprotective agents [88].

In the 2012 *EJMC* paper [89], we reported the neuroprotection capacity of (*Z*)-*N*-(2-bromo-5-hydroxy-4-methoxybenzylidene)-2-methylpropan-2-amine oxide (3) (Figure 3) on rat cortical neurons in culture, under OGD conditions [89]. Nitrone 3 was analyzed in our 2011 *BMC* paper [87], and, although the determined RSA(%) values in the DPPH test (31.1%), ORAC method (5.58 μM Trolox/μM compound), and in the benzoic assay for hydroxyl radical scavenging (96.3%) were not the best among the nitrones analyzed in that paper [87], we selected nitrone 3 for in depth neuroprotection analysis [89].

**Figure 5:**
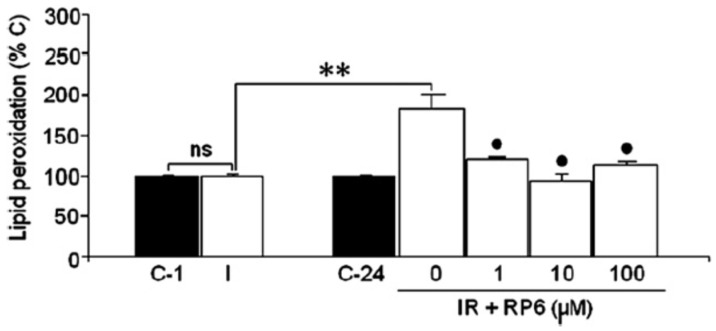
Lipid peroxidation with OGD treatment. Action of nitrone 3 during reperfusion. C-1 = control 1 h, C-24 = control at 24 h, I = 1 h OGD treatment, IR = 1 h OGD plus 24 h reperfusion in absence (0) or presence of 1, 10, and 100 mM nitrone **3**. Results are given as percentages of their respective control. ns = no significant, ** = *p* < 0.005. (•) represents the statistical signification between cortical neurons subjected to reperfusion in absence and presence of nitrone 3. Reproduced with permission from ref. [89]. Copyright 2012. Elsevier.

**Figure 6:**
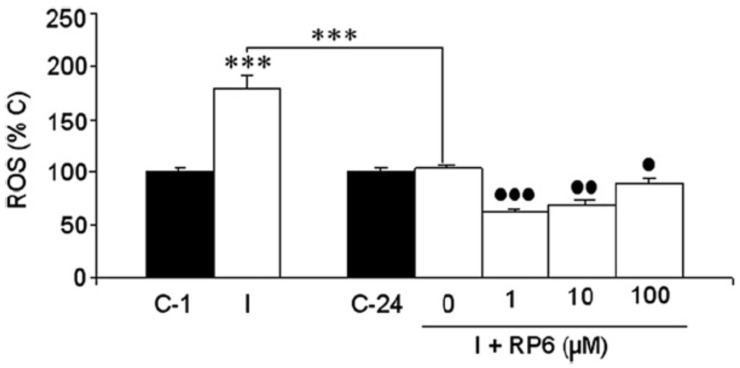
ROS generation by OGD treatment. Action of nitrone 3 during reperfusion period. C-1 = control 1 h, C-24 = control 24 h, I = 1 h OGD treatments, IR = 1 h OGD plus 24 h reperfusion in absence (0) or presence of 1, 10, and 100 mM of nitrone 3. Results are given as percentages of their respective control. *** = *p* < 0.005. (•), (••), (•••) represents the statistical signification between cortical neurons subjected to reperfusion in absence and presence of nitrone 3. Reproduced with permission from ref. [89]. Copyright 2012. Elsevier.

**Figure 7:**
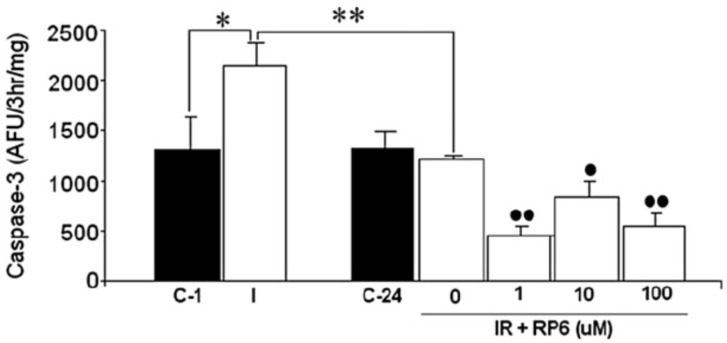
Effect of OGD on caspase-3 activity. Action of reperfusion in absence or presence of nitrone 3. C-1 = control, C-24 = control at 24 h, I = 1 h OGD, IR = 1 h OGD plus 24 h reperfusion in absence (0) or presence of 1, 10, and 100 mM of nitrone 3. Results are given as UAF/3 h/mg protein. * = *p* < 0.005, ** = *p* < 0.005. (•), (••) represents the statistical signification between cortical neurons subjected to reperfusion in absence and presence of nitrone **3**. Reproduced with permission from ref. [89]. Copyright 2012. Elsevier.

Thus, following the usual methods, cortical neurons in culture were treated during 1 h with OGD. After, they were placed under normal conditions during 24 h (reperfusion) in the absence and presence of nitrone 3. As expected, after 1 h of OGD treatment, a loss of respiratory ability, LDH release, caspase-3 activation, and ROS formation were induced. After 24 h of reperfusion, in the absence of nitrone 3, necrosis was observed and the lipid peroxidation increased, but the ROS formation and the caspase-3 activation were reversed. However, nitrone 3 administered during the reperfusion process was able to protect cortical neurons against necrotic death, lipid peroxidation (Figure 5), ROS formation (Figure 6), and caspase-3 activation (Figure 7) [89].

To sum up, the results obtained in the 2012 *EJMC* paper [89] show that nitrone 3 protects neurons against ROS generation, lipid peroxidation levels, LDH release, and mitochondrial membrane potential alteration when administered during reperfusion after the OGD damage. Consequently, these results confirm that nitrone 3 protects cells against ischemia injury produced during the reoxygenation and could be a potential drug for the therapy of ictus. Finally, it is interesting to note that, as shown in Table 1 and the antioxidant results reported in the 2012 *JMC* [88] paper, nitrone 3 showed no capacity to inhibit LOX but modest power to inhibit LP (67%), trap ^•^OH (72%), O_2_*^•^* (11%), and NO (2%) [88].

### 3.3. Neuroprotection of PBN-Indanonitrones 21–26

**Figure 8:**
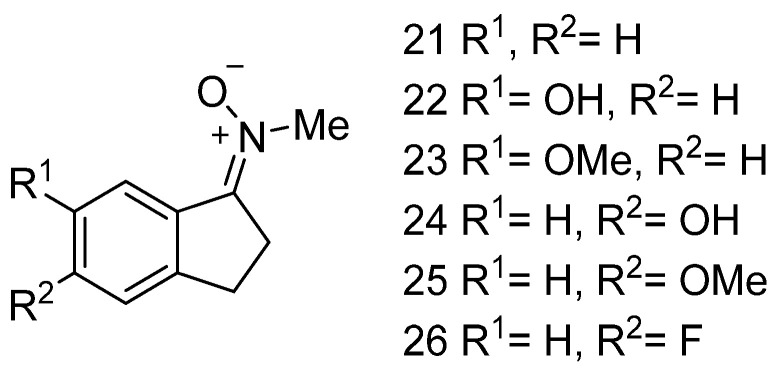
Structures of PBN-indanonitrones 21–26.

In our 2019 *BC* paper [90], six rigid PBN-related indanonitrones 21–26 (Figure 8) were designed in order to ascertain the effect that preventing the free conformational freedom by inserting a five membered–ring system may have on their neuroprotective and antioxidant capacities. We synthesized them as usual (see above), starting from the corresponding commercially available indanones and *N*-methylhydroxylamine. The neuroprotective capacity of these indanonitrones 21–26 (Figure 8) was tested in vitro, under OGD conditions, in SH-SY5Y human neuroblastoma cell cultures. As a result, we identified indanonitrones 21, 23, and 24 as the most neuroprotective agents. Among these, indanonitrone 24, bearing an hydroxyl group at C5, was not only the most potent (EC_50_ = 6.64 ± 0.28 μM) but also the most balanced nitrone, as it showed antioxidant activity in three experiments [LOX (100 μM), APPH (51%), DPPH (36.5%)]; it is clearly a more potent antioxidant agent than nitrone PBN. Consequently, we identified (*Z*)-5-hydroxy-*N*-methyl-2,3-dihydro-1*H*inden-1-imine oxide (24) as the most interesting nitrone in this family of nitrones, confirming that rigid PBN-related indanonitrones, such as 24, have more neuroprotective and antioxidant powers than standard PBN [90].

### 3.4. Neuroprotection of PBN-Derived Homo-Bis-Nitrones 27–35

**Figure 9:**
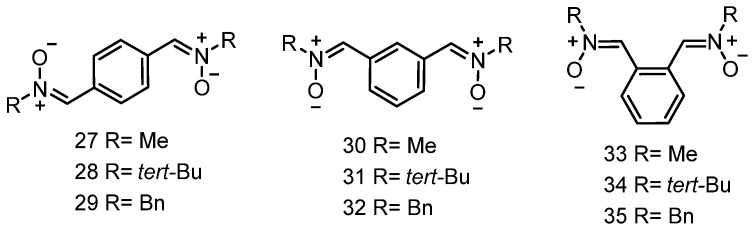
Structures of homo-bis-nitrones HBNs27–35.

In the 2020 *SR* paper [91], we reported the synthesis, antioxidant power, and neuroprotective properties of nine homo-bis-nitrones HBNs27–35 (Figure 9) as PBN analogues for stroke therapy. In vitro neuroprotection studies of HBNs27–35 against oligomycin A/rotenone (O/R) and in an oxygen-glucose-deprivation model of ischemia in human neuroblastoma cell cultures indicated that (1*Z*,1′*Z*)-1,1′-(1,3-phenylene)bis(*N*-benzylmethanimine oxide) (HBN32) (Figure 9), bearing two *N*-benzyl motifs attached to the nitrone nitrogen in relative *meta* position around the benzene core, was a potent neuroprotective agent that prevented the decrease in neuronal metabolic activity (EC_50_ = 1.24 ± 0.39 μM) as well as necrotic and apoptotic cell death. HBN32 showed a strong hydroxyl radical scavenger power (81%) and the capacity to decrease superoxide production in human neuroblastoma cell cultures (maximal activity = 95.8 ± 3.6%), values significantly superior to the neuroprotective and antioxidant properties of the parent PBN. Calculated physicochemical and ADME properties confirmed HBN32 as a hit-agent showing suitable drug-like properties.

The contribution of HBN32 to brain damage prevention was confirmed in a permanent MCAO setting by assessing infarct volume outcome 48 h after stroke in drug administered experimental animals, which provided evidence of a significant reduction of the brain lesion size, strongly suggesting that HBN32 is a potential neuroprotective agent against stroke [91].

### 3.5. Neuroprotection of PBN-Derived Hetero-Bis-Nitrones 36–44

**Figure 10:**
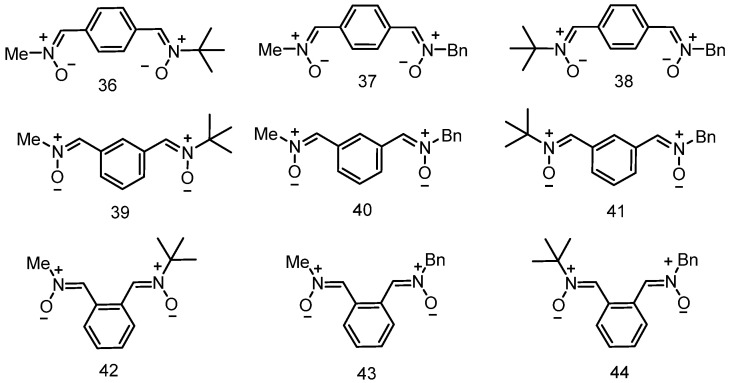
Structures of hetero-bis-nitrones hBNs36–44.

These interesting results prompted us to investigate related bis-PBN-nitrones, designed by incorporating mixed *N*-alkyl (methyl, *tert*-butyl, benzyl) motifs attached to the nitrone nitrogen in relative *ortho*, *meta*, and *para* position around the same benzene core, an analysis that resulted in the nine hetero-bis-nitrones hBNs36–44 (Figure 10) [92] as new PBN analogues. The synthesized hBNs36–44 were evaluated for their antioxidant activity using different in vitro techniques, while they were also tested as inhibitors of soybean LOX, an indication of their anti-inflammatory effect. Nitrone hBN44, bearing *N*-*tert*-butyl and *N*-benzyl motifs attached to the nitrone nitrogen in a relative *ortho* position around the benzene core, was the most potent antioxidant, presenting the highest anti-lipid peroxidation and hydroxyl radicals scavenging activities as well as the highest LOX inhibition. In silico calculations reveal that hBN44 follows Lipinski’s rule of five and that the molecule is theoretically able to penetrate the brain. All these results led us to propose hBN44 as a potent new antioxidant nitrone with a promising neuroprotective profile for potential use in the stroke therapy.

### 3.6. Neuroprotection of PBN-Derived Homo-Tris-Nitrones 45–47

**Figure 11:**
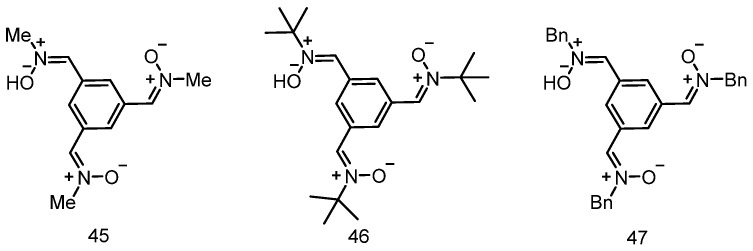
Structures of PBN-nitrones HTNs45–47.

In our next contribution [93], we wondered whether the neuroprotective effect of these polysubstituted PBN-derived nitrones might be additive, and, consequently, we designed the homo-tris-nitrones HTNs45–47 (Figure 11). Thus, we reported the synthesis, antioxidant, and neuroprotective powers of HTNs45–47, working on the hypothesis that the incorporation of a third nitrone motif into our previously identified homo-bis-nitrone HBN32 (Figure 9) would result in improved and stronger neuroprotection [8]. The neuroprotection of HTNs45–47, measured against O/R, showed that HTN46, bearing three *N*-*tert*-butyl motifs attached to the nitrone nitrogen in a relative *meta* position around the benzene core, was the best neuroprotective agent at a lower dose (EC_50_ = 51.63 ± 4.32 μM), being similar in EC_50_ and maximal activity to PBN. The results of neuroprotection in an in vitro oxygen-glucose-deprivation model showed that HTN46 was the most powerful (EC_50_ = 87.57 ± 3.87 μM) but was ~1.7-fold less potent than PBN. HTN47, bearing three *N*-benzyl motifs attached to the nitrone nitrogen in a relative *meta* position around the benzene core, had a very good antinecrotic (IC_50_ = 3.47 ± 0.57 μM), antiapoptotic, and antioxidant (EC_50_ = 6.77 ± 1.35 μM) profile. In summary, HTNs46 and 47 are attractive neuroprotective agents, clearly exceeding PBN [93].

### 3.7. Neuroprotection of PBN-Derived 1,1′-Biphenylnitrones 48–53

**Figure 12:**
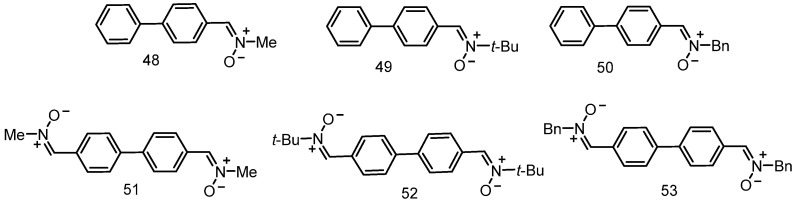
Structures of PBN-nitrones the BPNs48–53.

In 2021, we reported the synthesis, neuroprotection in in vitro ischemia models, and antioxidant activity of 1,1′-biphenylnitrones BPNs48–53 (Figure 12) as new PBN-analogues [94]. BPNs48–50 are mono-nitrone PBN-analogues prepared from commercially available [1,1′-biphenyl]-4-carbaldehyde, whereas BPNs51–53 are bis-nitrone PBN-analogues prepared from commercially available [1,1′-biphenyl]-4,4′-dicarbaldehyde [94]. The neuroprotection of BPNs48–53 was measured against O/R and in an oxygen-glucose-deprivation in vitro ischemia model in human neuroblastoma SH-SY5Y cells. Our results indicate that BPNs48–53 have better neuroprotective and antioxidant properties than PBN and quite similar neuroprotective and antioxidant properties to *N*-acetylcysteine, a well-known antioxidant agent. Among the nitrones studied, homo-bis-nitrone BPN53, bearing two *N*-*tert*-butyl radicals at the nitrone motif, has the best neuroprotective capacity (EC_50_ = 13.16 ± 1.65 and 25.5 ± 3.93 μM) against a reduction in metabolic activity induced by respiratory chain blockers and oxygen-glucose-deprivation in vitro ischemia model, respectively. It also has the highest anti-necrotic, anti-apoptotic, and antioxidant activities (EC_50_ = 11.2 ± 3.94 μM), measured by its capacity to reduce superoxide production in human neuroblastoma SH-SY5Y cell cultures, followed by mono-nitrone BPN50, with one *N*-benzyl radical, and BPN49, with only one *N*-*tert*-bujtyl substituent. The antioxidant power of BPN53 was also analyzed for its capacity to scavenge hydroxyl free radicals (82% at 100 μM), LOX inhibition, and inhibition of lipid peroxidation (68% at 100 μM). These results show that, although the number of nitrone groups improves the neuroprotection profile of these BPNs, the final effect is also dependent on the substituent that is being incorporated. Thus, BPNs bearing *N*-*tert*-butyl and *N*-benzyl groups show better neuroprotective and antioxidant properties than those substituted with methyl. All these results led us to propose homo-*bis*-nitrone BPN53 as the most well-balanced and interesting nitrone, based on its neuroprotective capacity in different neuronal models of oxidative stress and in vitro ischemia and its antioxidant power [94].

### 3.8. Neuroprotection of Polyfunctionalized PBN-Based Nitrones 54–65

**Figure 13:**
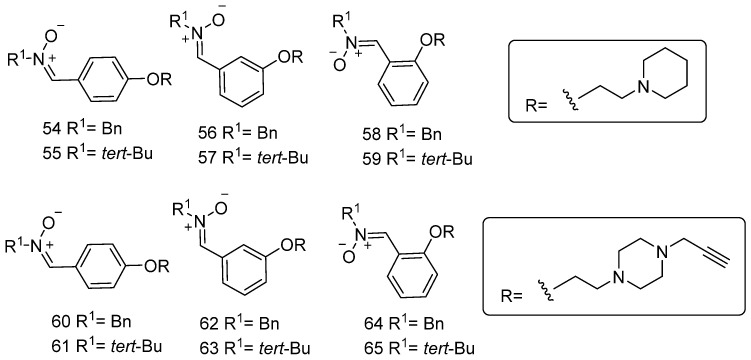
Structures of the polyfunctionalized PBN-based nitrones 54–65.

In one of the most recent contributions from our group in the search for new and neuroprotective PBN-nitrones [95], we recently described the design and synthesis of the polyfunctionalized PBN-derived *para-, meta*-, and *ortho*- substituted nitrones 54–65 (Figure 13), bearing piperidinepropoxy- and *N*-propargyl-piperazinepropoxy, reputed pharmacophore groups, to allow the inhibition of cholinesterase and monoamino oxidase enzymes by these nitrones. The final target is to identify neuroprotective multivalent PBN-nitrones for the combined therapy of AD and stroke. As a result of this investigation, we identified PBN-nitrone (*Z*)-*N*-benzyl-1-(2-(3-(piperidin-1-yl)propoxy)phenyl)methanimine oxide (58) (Figure 13), an *N*-benzylnitrone bearing the piperidinepropoxy motif in *ortho* postion, as the most potent antioxidant, with high ABTS^+^ scavenging activity (19%) and potent LOX inhibitory capacity (IC_50_ = 10 μM), selectively inhibiting human butyrylcholinesterase (IC_50_ = 3.46 ± 0.27 μM) and exhibiting a neuroprotective profile against the neurotoxicant okadaic acid in a neuronal damage model. Overall, these results pave the way for further in-depth analysis of the neuroprotection of nitrone 58 in in vitro and in vivo models of stroke [95] and, possibly, other neurodegenerative diseases in which oxidative stress is identified as a critical player.

### 3.9. Neuroprotection of PBN-Based Nitrones 66–97

**Scheme 2:**
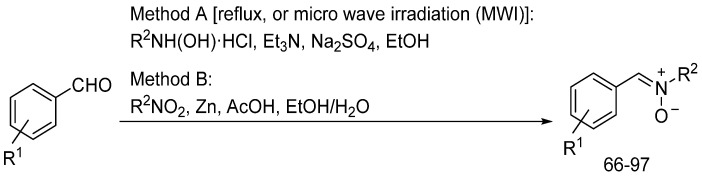
Synthesis of PBN-nitrones 66–97.

Finally, very recently, I reported the synthesis, antioxidant, and biological evaluation of the 32 monosubstituted α-arylnitrones, shown in Table 6, whose design was inspired by the structure of PBN, looking for novel chemical entities for CI treatment [96]. The synthesis of these PBN-nitrones 66–97 was carried out from modest to good yields by reacting easily-available, diversely-substituted benzaldehydes, incorporating electron-rich hydroxy, propargyloxy, and methoxy substituents, or electron-poor nitro, fluoro, and trifluoromethyl groups, with commercial or *N*-methyl, *N*-benzyl, *N*-*tert*-butyl, and *N*-phenyl hydroxylamines, as shown in Scheme 2.

**Figure 14:**
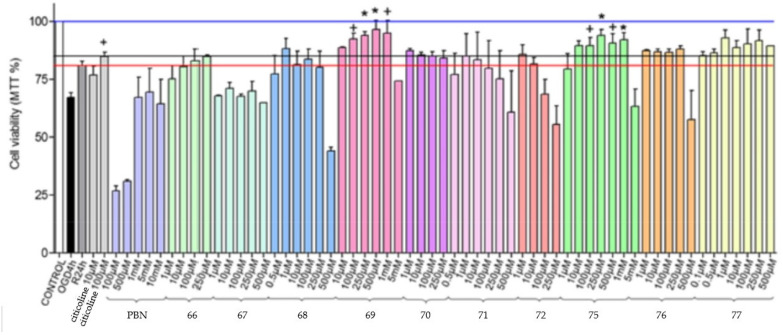
Effect of PBN, citicoline, and PBN-nitrones 66–72 and 75–77, on primary neuronal cultures subjected to OGD. Bar chart showing the percentage of cell viability at 24 h of recovery after 4 h OGD, either untreated (R24 h, 80.9%, dark grey bar, red line) or treated with different concentrations of novel PBN-nitrones 66–72 and 75–77, PBN, or citicoline 100 µM (84.9%, light grey bar, black line). The value induced by OGD at 4 h without recovery period is also indicated (OGD4 h, 67.3%, black bar). Cell viability corresponding to control cells (1.95 ± 0.09 AU) was considered as 100% (blue line). The values represent the average of three to seven independent experiments (error bars represent the SE). * *p* < 0.05 versus R24 h by one-way analysis of variance following Dunnett’s test, or ^+^ *p* < 0.05 by Student’s *t* test, when analysis of variance was significant. Statistical significances below R24 h value were not shown. Reproduced with permission from ref. [96]. Copyright 2024. Elsevier.

Next, the neuroprotective capacity of nitrones 66–97 was assessed in vitro on neurons under OGD conditions for 4 h. Then, compounds 66–97 (Table 6) and standards PBN and citicoline were incorporated in different doses. As shown in Figure 14, 4 h OGD resulted in high neuron death (67.3% vs. control 100%) but reverted after 24 h of recovery (80.9% vs. control 100%).

Regarding nitrones 66–77, and as shown in Figure 14, these compounds produce, at least at one of the doses investigated, less neuron death than the non-treated group (R24h, dark grey bar, red line). Note also that the substituted *N*-*tert*-butyl ligands 69 and 75 bearing 2-hydroxy and 2-*O*-propargylated groups, respectively, presented a good neuroprotective profile, showing better effect than standards PBN, NXY-059, and citicoline, for instance (Table 7). The same protocol was applied to the rest of PBN-nitrones 78–97, but without better results [96]. Not surprisingly, and from the antioxidant test, compounds 69 and 75 presented related ClogP and antioxidant capacity, strongly inhibiting LP (100%) and LOX (53 μM), being unable to trap 1,1-diphenyl-2-picrylhyrazyl and hydroxyl radicals or to loosen NO. Based on these attractive and promising results, we were prompted to analyze nitrones 69 and 75 in an appropriate in vivo CI model of transient global CI [96].

**Figure 15:**
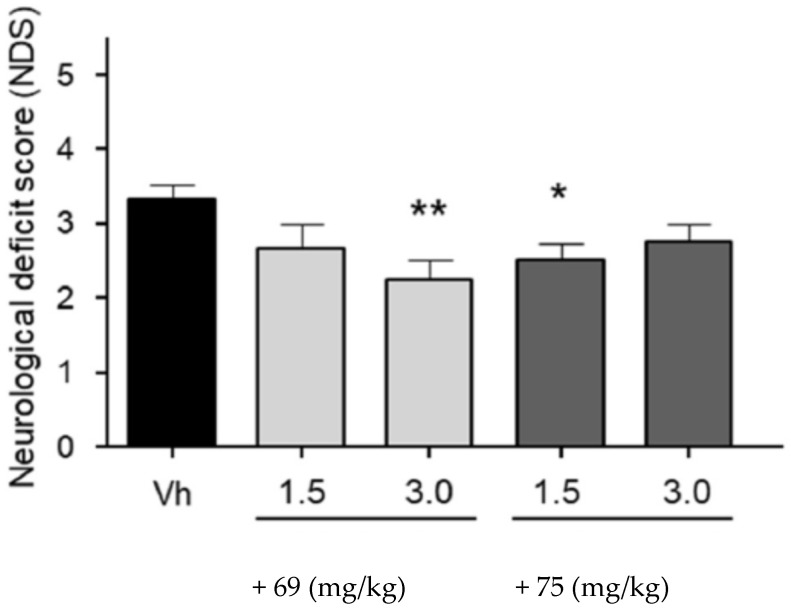
Neurodeficit score outcomes after CI in the dose–response study of 69 and 75. The bar graph shows the NDS in ischemic animals at 5 days of reperfusion after ischemia. Animals were treated with vehicle (Vh), 69 (1.5 and 3.0 mg/kg), or 75 (1.5 and 3.0 mg/kg) by an intravenous injection at the onset of reperfusion after cerebral ischemia. Data represent the mean of 6–12 independent animals per group, and the error bars indicate the SE. * *p* < 0.05 and ** *p* < 0.01, compared with vehicle by Dunn’s test after the Kruskal–Wallis test. Reproduced with permission from ref. [96]. Copyright 2024. Elsevier.

In this protocol, a short ischemia was produced, inducing neurodegeneration in the hippocampal CA1 area. Nitrones 69 and 75 were added in 1.5 and 3.0 mg/kg doses, amounts that result in 130–260 μM and 107–215 μM in blood for 69 and 75, respectively, taking into account that dose 250 μM was already neuroprotective, as shown above in the OGD test. Animals were submitted to CI for 15 min, treated with vehicle, 69, or 75, and studied after 5 days of RI, evaluating the NDS. As shown in Figure 15, and according to the NDS (3.3 ± 0.18), vehicle-treated RI animals were strongly affected by comparison with sham control animals (NDS = 0), whereas nitrones 69 (at 3.0 mg/kg) and 75 (at 1.5 mg/kg) significantly reduced NDS ((2.25 ± 0.25) and (2.75 ± 0.25, *p* < 0.05), respectively).

**Figure 16:**
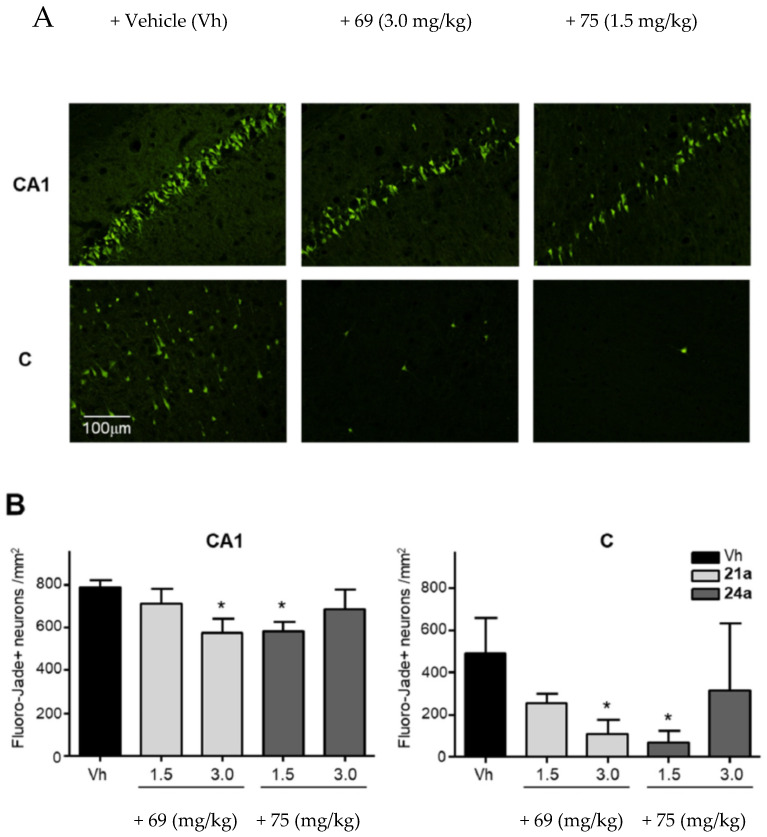
Compounds 69 and 75 prevent the neuronal death induced after transient CI. Ischemic animals were treated with 69 (1.5 or 3.0 mg/kg), 75 (1.5 or 3.0 mg/kg), or vehicle (Vh) by intravenous injection at the onset of reperfusion after CI. The neuroprotective effect was studied, assessing the ischemia-induced neuronal death at 5 days of reperfusion (R5d). Neuronal death was detected in brain sections stained with Fluoro-Jade B and visualized by fluorescence microscopy (in green). (**A**) Representative images of Fluoro-Jade B-stained brain sections of the hippocampal CA1 (CA1) and cortex (C) regions from untreated (Vh) and treated (69, 3.0 mg/kg and 75, 1.5 mg/kg) animals; (**B**) Fluoro Jade-positive neurons were counted in CA1 or C fields as described in the Methods section. Bar graphs represent the mean of 6–12 individual animals. Error bars indicate SE. * *p* < 0.05, compared with vehicle (Vh) by Dunnett’s test after ANOVA. Reproduced with permission from ref. [96]. Copyright 2024. Elsevier.

Following the same protocol as above, next, the ischemia-induced neuronal death in the hippocampal CA1 and cortical regions in brain sections was evaluated using Fluoro-Jade B staining. As shown in Figure 16A, the administration of nitrones 69 (3.0 mg/kg) and 75 (1.5 mg/kg) strongly reduced neuronal death in the CA1 area, producing neuroprotection, by comparison with the vehicle-treated control group—an effect that could also be noticed in the cerebral cortex (Figure 16B).

To sum up, nitrones 69 and 75 proved to be neuroprotective agents in in vivo models of CI, significantly decreasing neuronal death and apoptosis in the CA1 brain area. In fact, nitrone 69, according to the NDS, showed a better neuroprotective profile in the CA1 area than nitrone 75, although 75 induced superior neuroprotection in the cortical cortex at half the dose than nitrone 69, meaning it has a higher neuroprotective power. So, I concluded that, with the increased activity in the case of nitrone 69 and increased potency for nitrone 75, both ligands can be proposed as novel hit-molecules for further in-depth analysis targeted to CI therapy.

## 4. Summary and Outlook

Stroke is the biggest cause of adult disability and the second-highest cause of dementia, with no effective drugs having yet reached the clinic for its therapy. Blood reperfusion with the thrombolytic agent rtPA is the only pharmacologic treatment currently available for ischemic stroke, but it has significant therapeutic limitations. It is, thus, a priority to develop neuroprotective strategies able to preserve neurons from ischemic injury and, in this way, reduce brain damage and patient disability. A promising approach involves the rescue of the area of the penumbra surrounding the infarct—a region functionally silent but structurally intact.

In this account, we have reviewed the medicinal chemistry of the antioxidant and neuroprotective agent PBN, as well as the recently discovered PBN-derived nitrones from my laboratory, in order to update and summarize the newly reported developments on PBN-derived nitrones for stroke therapy. The presented results confirm that nitrones continue to offer opportunities for improvement and therapeutic efficacy.

The protective effect of PBN in the transient MCAO has been demonstrated, with PBN reducing both neuronal mortality and neuronal damage in the CA1 area of the hippocampus caused by ischemia. However, PBN fails to prevent postischemic CA1 damage in rats. As for focal cerebral ischemia, PBN significantly reduces cerebral infarction and decreases neurological deficit after ischemia using a rat model of persistent MCAO.

The new PBN-nitrones have created great expectations, appearing to be attractive compounds due to their strong antioxidant power and multi-target pharmacological activity, which enable them to target different events of the biochemical routes involved in the ischemic insult.

## Figures and Tables

**Table 1:** In vitro antioxidant activities for PBN-derived nitrones 1–13 ^a^ [87].

Nitrone	Structure	DPPH [RSA (%)] ^b^	ORAC ^d^	^•^OH [RSA (%)] ^e^
1	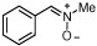	0.8 ± 0.7	0.0 ± 0.0	95.3 ± 0.8
PBN	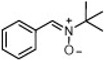	1.4 ± 0.9	0.0 ± 0.0	94.9 ± 1.0
2	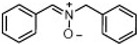	0.6 ± 0.3	0.01 ± 0.00	95.7 ± 0.5
3	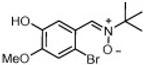	31.1 ± 2.1	5.58 ± 0.33	96.3 ± 0.5
4	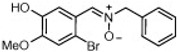	25.9 ± 1.8	7.36 ± 0.32	94.6 ± 0.9
5	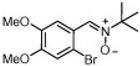	0.8 ± 0.9	0.03 ± 0.00	96.3 ± 0.8
6	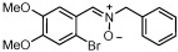	0.2 ± 0.2	0.03 ± 0.002	95.1 ± 1.3
7	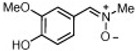	87.6 ± 1.2	3.35 ± 0.07	96.5 ± 0.7
8	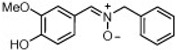	88.2 ± 1.0	2.93 ± 0.07	96.9 ± 0.7
9	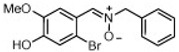	40.7 ± 2.5	6.40 ± 0.35	95.7 ± 1.0
10	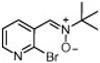	3.9 ± 3.8	0.01 ± 0.00	95.1 ± 1.1
11	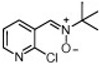	1.6 ± 1.5	0.01 ± 0.00	94.9 ± 1.3
12	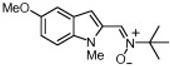	4.8 ± 1.8	7.98 ± 0.21	nd ^c^
13	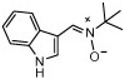	1.2 ± 1.1	3.69 ± 0.15	nd ^c^

^a^ Data are expressed as mean values (±SEM) of at least three different experiments in quadruplicate. ^b^ Determined at 0.5 mM. ^c^ nd, not determined. ^d^ micromol trolox/micromol compound. ^e^ Benzoic acid methods: determined at 50 μM. Reproduced with permission from ref. [87]. Copyright 2011. Elsevier.

**Table 2:** In vitro antioxidant activities for PBN-derived nitrones 1–13 [88].

Nitrone	LOX (%) ^d^	AAPH (%) ^e^ (min) ^f^	HO^• g,h^	O_2_^•^ (%) ^i^	NO (%) ^k,(m)^	clog*P*
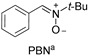	83	No (0)	90	15	2 (4.5)	2.56
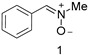	22	5 (1)	77	8	No (No)	1.32
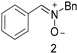	No	74 (25.2)	97	24	nd ^l^ (No)	3.09
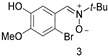	No	67 (7.2)	72	11	2 (3)	2.83
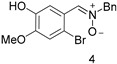	No	89 (84.6)	No	6	2 (2)	3.36
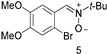	No	47 (0.52)	90	15	1 (2)	3.15
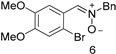	21	No (53.7)	No	16	1 (1)	3.68
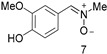	59	90 (18)	No	11	7 (8)	0.5
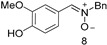	No	90 (88.2)	52	No	9 (10)	2.67
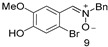	24	87 (41.8)	78	3	5 (6)	3.36
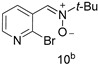	88	40 (8)	93	10	4 (5.5)	2.01
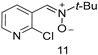	59	37 (29.8)	30	5	2 (4)	1.86
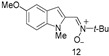	31	80 (43.8)	79.5	5	3 (5)	3.07
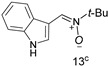	42	57 (21.6)	59	15	3 (4.5)	2.57
NDGA	40					
Caffeic acid				15		
Trolox		63 (62.1)	73			
SNP					54 (58)	

^a^ 0 at 0.01 mM, 0 at 0.05 mM); ^b^ 0 at 0.01 mM, 0 at 0.05 mM; ^c^ 0 at 0.01 mM, 0 at 0.05 mM. These results concern the % LOX inhibition; ^d^ inhibition at 0.1 mM; ^e^ inhibition at 0.1 mM; ^f^ induction time t_inh_ produced by the tested nitrones; ^g^ inhibition at 0.1 mM; ^h^ these results concern the ability of nitrones to compete with DMSO for hydroxyl radicals; ^i^ 0.1 mM; ^k^ 0.1 mM + 1 mM cysteine; ^l^ nd: not determined; ^m^ nitrate reductase/NADPH system.

**Table 3:** In vitro antioxidant activities for nitrones 14–20 [88].

Nitrone	LOX (%) ^a^	AAPH (%) ^b^ (min) ^c^	DPPH (RSA)(%) ^d^	OH ^f^	O_2_^•^ (%) ^g^	clog*P*
14	65	No (0)	0.9	60	29	3.44
15	No	61 (83)	81.9	77	8	5.87
16	No	9 (17.4)	78.6	No	No	5.34
17	19	No (0)	0.6	98	19	3.04
18	21	64 (46)	nd ^h^	100	No	3.57
19	57	49 (47)	0.4	54	2	3.29
20	30	81 (36.6)	Nd ^h^	98	No	3.83
Caffeic acid					15	
Curcumin			35.0 ^e^			
Quercetin			94.2 ^e^			
Trolox		63 (62.1)				

^a^ Determined at 0.1 mM; ^b^ determined at t 0.1 mM; ^c^ induction time t_inh_ produced by the tested nitrones; ^d^ determined at 0.5 mM; ^e^ determined at 15 μM; ^f^ at 0.1 mM; ^g^ at 0.1 mM; ^h^ nd: not determined.

**Table 4:** Neuroprotective effect for nitrones 14 and 20 in neuronal cultures exposed to OGD.

Nitrone (Concentration)	Neuroprotection (%)
14 (100 μM)	101.3 ± 3.3 ***
14 (250 μM)	81.2 ± 5.0 ***
20 (10 μM)	77.6 ± 3.1 ***
20 (100 μM)	83.0 ± 2.7 ***
PBN (5 mM)	−13.4 ± 1.9 ***
Citicoline (100 μM)	53.2 ± 1.8

Neuroprotection was defined as the percentage to reach the control value, defined as 100%, from R24 h value, defined as 0%. *** *p* < 0.001 compared to citicoline, by Student’s *t* test.

**Table 5:** Neuroprotective effect for nitrones 14, 15, and 17–20 against necrotic cell death in neuronal cultures exposed to OGD.

Nitrone	Concentration (μM)	Neuroprotection against Necrotic Cell Death (%)
14	250	70.08 ± 7.52 *
15	100	53.80 ± 6.01
15	250	56.95 ± 10.6
17	250	79.21 ± 19.55
18	1	47.48 ± 3.33
19	100	55.57 ± 12.51
20	10	45.37 ± 2.41
PBN	100	67.82 ± 4.70 **
	250	73.20 ± 5.83 **
Citicoline	100	61.26 ± 8.26

Neuroprotection against necrotic cell death was defined as the percentage to reach the control C value (defining as 100% neuroprotection the difference between R24 h and C percentages). * *p* < 0.05, ** *p* < 0.01 compared to 50% neuroprotection, by one-sample Student’s *t* test.

**Table 6:** Synthesis and structure of PBN-nitrones 66–97.

R^1^	R^2^	Method	Time	Nitrone	Yield (%)
H	Bn	A (reflux)	1 d	66	68
H	Me	A (MWI)	20 min	67	61
H	Ph	B	2 h	68	57
2-OH	*t*-Bu	A (rt)	2 d	69	86
2-OH	Bn	A (reflux)	5 d	70	99
2-OH	Me	A (reflux)	1 d	71	73
2-OH	Ph	B	4 d	72	86
3-OH	*t*-Bu	A (MWI)	9 h	73	30
4-OH	*t*-Bu	A (MWI)	5 h	74	71
2-O-CH_2_-C≡CH	*t*-Bu	A (rt)	4 d	75	77
2-O-CH_2_-C≡CH	Bn	A (rt)	7 d	76	62
2-O-CH_2_-C≡CH	Me	A (rt)	4 d	77	60
3-O-CH_2_-C≡CH	*t*-Bu	A (rt)	5 d	78	30
4-O-CH_2_-C≡CH	*t*-Bu	B	5 d	79	28
2-OMe	*t*-Bu	A (reflux)	1 d	80	81
3-OMe	*t*-Bu	A (reflux)	4 d	81	72
4-OMe	*t*-Bu	A (reflux)	3 d	82	79
2-NO_2_	*t*-Bu	A (MWI)	6 h	83	49
2-NO_2_	Bn	A (MWI)	50 min	84	50
3-NO_2_	*t*-Bu	A (MWI)	9 h	85	70
3-NO_2_	Bn	A (MWI)	2 h	86	68
4-NO_2_	*t*-Bu	A (reflux)	4 d	87	91
4-NO_2_	Bn	A (MWI)	1 h 15 min	88	69
2-F	*t*-Bu	A (reflux)	1 d	89	66
3-F	*t*-Bu	A (MWI)	1 h	90	82
3-F	Bn	A (MWI)	3.5 h	91	89
4-F	*t*-Bu	A (reflux)	2 d	92	84
2-CF_3_	*t*-Bu	A (reflux)	2 d	93	70
2-CF_3_	Bn	A (MWI)	1 h 45 min	94	91
3-CF_3_	*t*-Bu	A (MWI)	7 h 30 min	95	64
3-CF_3_	Bn	A (MWI)	1 h	96	57
4-CF_3_	*t*-Bu	A (reflux)	1 d	97	84

**Table 7:** Neuroprotective effect for PBN-nitrones 69 and 75 in neuronal cultures subjected to OGD 4 h.

Nitrone	Conc.	Neuroprotection (%) ^a^
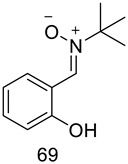	100 µM	60.8 ± 1.6
	250 µM	69.0 ± 1.3 *
	500 µM	82.7 ± 3.2 **
	1 mM	74.3 ± 4.3 *
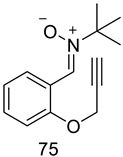	100 µM	46.1 ± 1.3
	250 µM	72.2 ± 2.1 **
	500 µM	48.6 ± 1.8
	1 mM	67.9 ± 1.9
PBN	5 mM	−59.4 ± 8.8
NXY-059	250 µM	56.8 ± 2.5
Citicoline	100 µM	21.1 ± 0.4

^a^ Neuroprotection value defined as the percentage to reach the control value, defined as 100%, from R24h value (80.9%), defined as 0%. Data (mean ± SE) were obtained from Figure 3 and Figure 4. * *p* < 0.1 and ** *p* < 0.01 compared to 250 µM NXY-059 by ANOVA and Dunnett’s test. Statistical significances of the data lower than 250 µM NXY-059 value were not shown.

## Data Availability

Data are contained within the article.

## Abbreviations

Adenosine diphosphate (ADP); Area under the concentration (AUC); Brain-derived neurotrophic factor (BDNF); *tert*-Butylhydroperoxide (*t*-BHP); 3-*n*-Butylphthalide (NBP); Cerebellum granular neurons (CGNs); Cerebral ischemia (CI); 3-(4,5-Dimethylthiazol-2-yl)-2,5-diphenyltetrazolium bromide (MTT); 2,2-Diphenyl-1-picrylhydrazyl (DPPH); Edaravone (Eda); Glutathione (GSH); Hydrogen peroxide (H_2_O_2_); 6-Hydroxydopamine (6-OHDA); Hydroxyl radical (^.^OH); Intraperitoneal (ip); Intravenous (iv); Malondialdehyde (MDA); Methyl-4-phenyl-1,2,3,6-tetrahydropyridine (MPTP); 1-Methyl-4-phenylpyridinium (MPP^+^); Middle cerebral artery occlusion (MCAO); Multitarget small molecule (MSM); Middle cerebral artery occlusion (MCAO); Neurological deficit score (NDS); Oxidative damage (OD); Oxygen−glucose deprivation (OGD); Oxygen Radical Absorbance Capacity (ORAC); Oxidative stress (OS); Parkinson’s disease (PD); Permanent MCAO (pMCAO); Peroxyl radical trapping (TRAP); α-Phenyl-*N-tert*-butylnitrone (PBN); Reactive nitrogen species (RNS); Reactive oxygen species (ROS); Recombinant tissue plasminogen activator (rtPa); Superoxide anion radical (O_2_^−^); Superoxide dismutase (SOD); Thiobarbituric acid reactive substance (TBARS); 2,3,5-Triphenyltetrazolium chloride (TTC).

## References

[1] Buccellato F.R., D’Anca M., Tartaglia G.M., Del Fabbro M., Scarpini E., Galimberti D. (2023). Treatment of Alzheimer’s Disease: Beyond Symptomatic Therapies. Int. J. Mol. Sci..

[2] Rosini M., Simoni E., Milelli A., Minarini A., Melchiorre C. (2014). Oxidative Stress in Alzheimer’s Disease: Are We Connecting the Dots?. J. Med. Chem..

[3] Olanow C.W. (1992). An Introduction to the Free Radical Hypothesis in Parkinson’s Disease. Ann. Neurol..

[4] Hardeland H. (2009). Neuroprotection by Radical Avoidance: Search for Suitable agents. Molecules.

[5] Pratico D., Delanty N. (2000). Oxidative Injury in Diseases of the Central Nervous System: Focus on Alzheimer’s Disease. Am. J. Med..

[6] Cummings J.L. (1994). Vascular Subcortical Dementias: Clinical Aspects. Dementia.

[7] Harman D. (1992). Role of Free Radicals in Aging and Disease. Ann. N. Y. Acad. Sci..

[8] Sack C.A., Socci D.J., Crandall B.M., Arendash G.W. (1996). Antioxidant Treatment with Phenyl-α-*tert*-butyl nitrone (PBN) Improves the Cognitive Performance and Survival of Aging Rats. Neurosci. Lett..

[9] Hammer B., Parker W.D., Bennett J.P. (1993). NMDA Receptors Increase OH Radicals in vivo by Using Nitric Oxide Synthase and Protein Kinase C. Neuroreport.

[10] Lafon-Cazal M., Culcasl M., Gaven F., Pietri S., Bockaert J. (1993). Nitric Oxide, Superoxide and Peroxynitrite: Putative Mediators of NMDA-nduced Cell Death in Cerebellar Granule Cells. Neuropharmacology.

[11] Coyle J.T., Puttfarcken P. (1993). Oxidative Stress, Glutamate, and Neurodegenerative Disorders. Science.

[12] Schulz J.B., Henshaw D.R., Siwek D., Jenkins B.G., Ferrante R.J., Cipolloni P.B., Kowall N.W., Rosen B.R., Beal M.F. (1995). Involvement of Free Radicals in Excitotoxicity in vivo. J. Neurochem..

[13] Mathers C.D., Boerma T., Fat D.M. (2009). Global and Regional Causes of Death. Br. Med. Bull..

[14] Rothman S.M., Olney J.W. (1986). Glutamate and the Pathophysiology of Hypoxic—Ischemic Brain Damage. Ann. Neurol..

[15] Lee J.M., Zipfel G.J., Choi D.W. (1999). The Changing Landscape of Ischaemic Brain Injury Mechanisms. Nature.

[16] Ito H., Kanno I., Ibaraki M., Hatazawa J., Miura S. (2003). Changes in Human Cerebral Blood Flow and Cerebral Blood Volume during Hypercapnia and Hypocapnia Measured by Positron Emission Tomography. J. Cereb. Blood Flow Metab..

[17] Choi D.W. (1996). Ischemia-induced Neuronal Apoptosis. Curr. Opin. Neurobiol..

[18] Hossmann K.A. (1994). Viability Thresholds and the Penumbra of Focal Ischemia. Ann. Neurol..

[19] Kontos H.A. (1989). Oxygen Radicals in CNS Damage. Chem. Biol. Interact..

[20] Zivin J.A., Fisher M., Degirolami U., Hemenway C.C., Stashak J.A. (1985). Tissue Plasminogen Activator Reduces Neurological Damage after Cerebral Embolism. Science.

[21] Adibhatla R.M., Hatcher J.F. (2008). Tissue Plasminogen Activator (tPA) and Matrix Metalloproteinases in the Pathogenesis of Stroke: Therapeutic Strategies. CNS Neurol. Disord. Drug Targets.

[22] Paschen W. (2000). Role of Calcium in Neuronal Cell Injury: Which Subcellular Compartment is Involved?. Brain Res. Bull..

[23] Niizuma K., Yoshioka H., Chen H., Kim G.S., Jung J.E., Katsu M., Okami N., Chan P.H. (2010). Mitochondrial and Apoptotic Neuronal Death Signaling Pathways in Cerebral Ischemia. Biochim. Biophys. Acta.

[24] Chan P.H. (2001). Reactive Oxygen Radicals in Signaling and Damage in the Ischemic Brain. J. Cereb. Blood Flow Metab..

[25] Iadecola C., Alexander M. (2001). Cerebral Ischemia and Inflammation. Curr. Opin. Neurol..

[26] Lo E.H., Moskowitz M.A., Jacobs T.P. (2005). Exciting, Radical, Suicidal: How Brain Cells Die after Stroke. Stroke.

[27] Bailly C., Hecquet P.-E., Kouach M., Thuru X., Goossens J.-F. (2020). Chemical Reactivity and Uses of 1-Phenyl-3-methyl-5-pyrazolone (PMP), also known as Edaravone. Bioorg. Med. Chem..

[28] Marco-Contelles J., Zhang Y. (2020). From Seeds of *Apium graveolens* Linn. to a Cerebral Ischemia Medicine: The Long Journey of 3-*n*-Butylphthalide. J. Med. Chem..

[29] Villamena F.A., Das A., Nash K.M. (2012). Potential Implication of the Chemical Properties and Bioactivity of Nitrone Spin Traps for Therapeutics. Future. Med. Chem..

[30] Marco-Contelles J. (2020). Recent Advances on Nitrones Design for Stroke Treatment. J. Med. Chem..

[31] Floyd R.A., Neto H.C.C.F., Zimmerman G.A., Hensley K., Towner R.A. (2013). Nitrone-based Therapeutics for Neurodegenerative Diseases: Their Use alone or in Combination with Lanthionines. Free Radic. Biol. Med..

[32] Floyd R.A., Towner R.A., He T., Hensley K., Maples K.R. (2011). Translational Research Involving Oxidative Stress and Diseases of Aging. Free Radic. Biol. Med..

[33] Costa D.S.S., Martino T., Magalhães F.C., Justo G., Coelho M.G.P., Barcellos J.C.F., Moura V.B., Costa P.R.R., Sabino K.C.C., Dias A.G. (2015). Synthesis of *N*-Methylarylnitrones Derived from Alkyloxybenzaldehydes and Antineoplastic Effect on Human Cancer Cell Lines. Bioorg. Med. Chem..

[34] O’Collins V.E., Donnan G.A., Macleod M.R., Howells D.W. (2013). Hypertension and Experimental Stroke Therapies. J. Cereb. Blood Flow Metab..

[35] Datta P.K., Reddy S., Sharma M., Lianos E.A. (2006). Differential nephron HO-1 expression Following Glomerular Epithelial Cell Injury. Nephron Exp. Nephrol..

[36] Mandal M.N., Moiseyev G.P., Elliott M.H., Kasus-Jacobi A., Li X., Chen H., Zheng L., Nikolaeva O., Floyd R.A., Ma J.-X. (2011). Alpha-phenyl-*N-tert*-Butylnitrone (PBN) Prevents Light-induced Degeneration of the Retina by Inhibiting RPE65 Protein Isomerohydrolase Activity. J. Biol. Chem..

[37] Ewert D., Hu N., Du X., Li W., West M.B., Choi C.-H., Floyd R., Kopke R.D. (2017). HPN-07, a Free Radical Spin Trapping Agent, Protects against Functional, Cellular and Electrophysiological Changes in the Cochlea Induced by Acute Acoustic Trauma. PLoS ONE.

[38] Varela-Nieto I., Murillo S., Rodríguez-de la Rosa L., Oset Gasque M.J., Marco-Contelles J. (2021). Use of Radical Oxygen Species Scavenger Nitrones to Treat Oxidative Stress-mediated hearing Loss: State of the Art and Challenges. Front. Cell. Neurosci..

[39] Rosselin M., Poeggeler B., Durand G. (2017). Nitrone Derivatives as Therapeutics: From Chemical Modification to Specific-targeting. Curr. Top. Med. Chem..

[40] Firuzi O., Miri R., Tavakkoli M., Saso L. (2011). Antioxidant Therapy: Current Status and Future Prospects. Curr. Med. Chem..

[41] Oliveira C., Benfeito S., Fernandes C., Cagide F., Silva T., Borges F. (2018). NO and HNO Donors, Nitrones, and Nitroxides: Past, Present, and Future. Med. Res. Rev..

[42] Floyd R.A., Kopke R.D., Choi C.-H., Foster S.B., Doblas S., Towner R.A. (2008). Nitrones as Therapeutics. Free Radic. Biol. Med..

[43] Croitoru M.D., Ibolya F., Pop M.C., Dergez T., Mitroi B., Dogaru M.T., Tőkes B. (2011). Nitrones Are Able to Release Nitric Oxide in Aqueous Environment under Hydroxyl Free Radical Attack. Nitric Oxide.

[44] Gothelf K.V., Jorgensen K.A. (1998). Asymmetric 1,3-Dipolar Cycloaddition Reactions. Chem. Rev..

[45] Durand G., Choteau F., Pucci B., Villamena F.A. (2008). Reactivity of Superoxide Radical Anion and Hydroperoxyl Radical with alpha-Phenyl-*N-tert*-butylnitrone (PBN) Derivatives. J. Phys. Chem. A.

[46] Cao X., Phillis J.W. (1994). α-Phenyl-*tert*-butyl-nitrone Reduces Cortical Infarct and Edema in Rats Subjected to Focal Ischemia. Brain Res..

[47] Durand G., Villamena F.A. (2013). Synthetic Antioxidants. Molecular Basis of Oxidative Stress.

[48] Lapchak P.A., Araujo D.M. (2002). Spin Trap Agents: A New Approach to Stroke Therapy. Drug News Perspect..

[49] Zausinger S., Hungerhuber E., Baethmann A., Hanns-Jürgen Reulen J., Schmid-Elsaesser R. (2000). Neurological Impairment in Rats after Transient Middle Cerebral Artery Occlusion: A Comparative Study under Various Treatment Paradigms. Brain Res..

[50] Novelli G.P., Angiolini P., Tani R., Consales G., Bordi L. (1986). Phenyl-T-butyl-nitrone is Active against Traumatic Shock in Rats. Free Rad. Res. Commun..

[51] Dehouck M.-P., Cecchelli R., Green A.R., Renftel M., Lundquist S. (2002). In vitro Blood-brain Barrier Permeability and Cerebral Endothelial Cell Uptake of the Neuroprotective Nitrone Compound NXY-059 in Normoxic, Hypoxic and Ischemic Conditions. Brain Res..

[52] Sun Y., Yu P., Zhang G., Wang L., Zhong H., Zhai Z., Wang L., Wang Y. (2012). Therapeutic Effects of Tetramethylpyrazine Nitrone in Rat Ischemic Stroke Models. J. Neurosci. Res..

[53] Robert A.F., Hema K.C., Ting H., Rheal T. (2011). Anti-cancer Activity of Nitrones and Observations on Mechanism of Action. Anti-Cancer Agents Med. Chem..

[54] Floyd R.A., Hensley K., Bing G. (2000). Evidence for Enhanced Neuro-inflammatory Processes in Neurodegenerative Diseases and the Action of Nitrones as Potential Therapeutics. J. Neural Transm. Suppl..

[55] Doggrell S.A. (2006). Nitrone Spin on Cerebral Ischemia. Curr. Opin. Investig. Drugs.

[56] Kuroda S., Tsuchidate R., Smith M.L., Maples K.R., Siesjo B.K. (1999). Neuroprotective effects of a Novel Nitrone, NXY-059, after Transient Focal Cerebral Ischemia in the Rat. J. Cereb. Blood Flow Metab..

[57] Macleod M.R., van der Worp H.B., Sena E.S., Howells D.W., Dirnagl U., Donnan G.A. (2008). Evidence for the Efficacy of NXY-059 in Experimental Focal Cerebral Ischaemia is Confounded by Study Quality. Stroke.

[58] Godínez-Rubí M., Rojas-Mayorquín A.E., Ortuño-Sahagún D. (2013). Nitric Oxide Donors as Neuroprotective Agents after an Ischemic Stroke-related Inflammatory Reaction. Oxid. Med. Cell. Longev..

[59] Matias A.C., Biazolla G., Cerchiaro G., Keppler A.T. (2016). α-Aryl-*N*-aryl Nitrones: Synthesis and Screening of a New Scaffold for Cellular Protection against an Oxidative Toxic Stimulus. Bioorg. Med. Chem..

[60] Minnerup J., Sutherland B.A., Buchan A.M., Kleinschnitz C. (2012). Neuroprotection for Stroke: Current Status and Future Perspectives. Int. J. Mol. Sci..

[61] Chen G., Griffin M., Poyer J.L., McCay P.B. (1990). HPLC Procedure for the Pharmacokinetic Study of the Spin-trapping Agent, alpha-Phenyl-N-tert-butyl Nitrone (PBN). Free Radic. Biol. Med..

[62] Saito K., Yoshioka H., Cutler R.G. (1998). A Spin Trap, *N*-*tert*-Butyl-*α*-phenylnitrone Extends the Life Span of Mice. Biosci. Biotech. Biochem..

[63] Saito K., Yoshioka H., Kazama S., Cutler R.G. (1998). Release of Nitric Oxide from a Spin Trap, *N-tert*-Butyl-α-phenylnitrone, under Various Oxidative Conditions. Biol. Pharm. Bull..

[64] Inanami O., Kuwabara M. (1995). α-Phenyl *N-tert*-butyl Nitrone (PBN) Increases the Cortical Cerebral Blood Flow by Inhibiting the Breakdown of Nitric Oxide in Anesthetized Rats. Free Rad. Res..

[65] Phillis J.W., Clough-Helfman C. (1990). Protection from Cerebal Ischemic Injury in Gerbils with the Spin Trap Agent *N-tert*-Butyl-aphenylnitrone (PBN). Neurosci. Lett..

[66] Clough-Helfman C., Phillis J.W. (1991). The Free Radical Trapping Agent *N-tert*-Butylalpha-phenylnitrone (PBN) Attenuates Cerebral Ischaemic Injury in Gerbils. Free Rad. Res. Commun..

[67] Sen S., Phillis J.W. (1993). alpha-Phenyl-*tert*-butyl-nitrone (PBN) Attenuates Hydroxyl Radical Production During Ischemia-reperfusion Injury of Rat Brain: An EPR Study. Free Rad. Res. Commun..

[68] Yue T.-L., Gu J.-L., Lysko P.G., Cheng H.-Y., Barone F.C., Feuerstein G. (1992). Neuroprotective Effects of Phenyl-*t*-butyl-nitrone in Gerbil Global Brain Ischemia and in Cultured Rat Cerebellar Neurons. Brain Res..

[69] Zhao Q., Pahlmark K., Smith M.L., Siesjö B.K. (1994). Delayed treatment with the Spin Trap alpha-Phenyl-*N-tert*-butyl Nitrone (PBN) Reduces Infarct Size Following Transient Middle Cerebral Artery Occlusion in Rats. Acta Physiol. Scand..

[70] Folbergrova J., Zhao Q., Katsura K., Siesjö B.K. (1995). *N-tert*-Butyl-alphaphenylnitrone Improves Recovery of Brain Energy State in Rats Following Transient Focal Ischemia. Proc. Natl. Acad. Sci. USA.

[71] Pahlmark K., Siesjö B.K. (1996). Effects of the Spin Trap α-Phenyl-*N-tert*-butylnitrone (PBN) in Transient Forebrain Ischemia in the Rat. Acta Physiol. Scand..

[72] Floyd R.A. (1990). Role of Oxygen Free Radicals in Carcinogenesis and Brain Ischemia. FASEB J..

[73] Oliver C.N., Starke-Reed P.E., Stadtman E.R., Liu G.J., Carney J.M., Floyd R.A. (1990). Oxidative Damage to Brain Proteins, Loss of Glutamine Synthetase Activity, and Production of Free Radicals during Ischemia/Reperfusion-induced Injury to Gerbil Brain. Proc. Natl. Acad. Sci. USA.

[74] Schulz J.B., Panahian N., Chen Y.I., Beal M.F., Moskowitz M.A., Rosen B.R., Jenkins B.G. (1997). Facilitation of Postischemic Reperfusion with a-PBN: Assessment Using NMR and Doppler Flow Techniques. Am. J. Physiol..

[75] Aronowski J., Strong R., Grotta J.C. (1997). Reperfusion Injury: Demonstration of Brain Damage Produced by Reperfusion after Transient Focal Ischemia in Rats. J. Cereb. Blood Flow Metab..

[76] Aronowski J., Grotta J., Waxham N.M. (1992). Ischemia Induced Translocation of Ca^2+^/Calmodulin-dependent Protein Kinase II: Potential Roles in Neuronal Damage. J. Neurochem..

[77] Aronowski J., Waxham M.N., Grotta J.C. (1992). Neuronal Protection and Preservation of Calcium/calmodulin-dependent Protein Kinase II and Protein Kinase C Activity by Dextrorphan Treatment in Global Ischemia. J. Cereb. Blood Flow Metab..

[78] Hiestand O.M., Haley B.E., Kindy M.S. (1992). Role of Calcium in Inactivation of Calcium/calmodulin Dependent Protein Kinase II after Cerebral Ischemia. J. Neurol. Sci..

[79] Pazos A.J., Green E.J., Busto R., McCabe P.M., Baena R.C., Ginsberg M.D., Globus M.Y.-T., Schneiderman N., Dietrich W.D. (1999). Effects of Combined Postischemic Hypothermia and Delayed *N-tert*-Butyl-α-phenylnitrone (PBN) Administration on Histopathological and Behavioral Deficits Associated with Transient Global Ischemia in Rats. Brain Res..

[80] Nakashima M., Niwa M., Iwai T., Uematsu T. (1999). Involvement of Free Radicals in Cerebral Vascular Reperfusion Injury Evaluated in a Transient Focal Cerebral Ischemia Model of Rat. Free Radic. Biol. Med..

[81] Schmid-Elsaesser R., Hungerhuber E., Zausinger S., Baethmann A., Reulen H.J. (2000). Neuroprotective Effects of the Novel Brain-penetrating Antioxidant U-101033E and the Spin-trapping Agent alpha-Phenyl-*N-tert*-butyl Nitrone (PBN). Exp. Brain Res..

[82] Yang Y., Li Q., Shuaib A. (2000). Neuroprotection by 2-h Postischemia Administration of Two Free Radical Scavengers, α-Phenyl-*N-tert*-butyl-nitrone (PBN) and *N-tert*-Butyl-(2-sulfophenyl)-nitrone (S-PBN), in Rats Subjected to Focal Embolic Cerebral Ischemia. Exp. Neurol..

[83] Li P.A., He Q.P., Nakamura L., Csiszar K. (2001). Free Radical Spin Trap α-Phenyl-*N*-*tert*-butyl-nitrone Inhibits Caspase-3 Activation and Reduces Brain Damage Following a Severe Forebrain Ischemic Injury. Free Rad. Biol. Med..

[84] Lapchak P.A., Chapman D.F., Zivin J.A. (2001). Pharmacological Effects of the Spin Trap Agents *N-t*-Butyl-phenylnitrone (PBN) and 2,2,6,6-Tetramethylpiperidine-*N*-oxyl (TEMPO) in a Rabbit Thromboembolic Stroke Model: Combination Studies with the Thrombolytic Tissue Plasminogen Activator. Stroke.

[85] Christensen T., Bruhn T., Diemer N.H. (2003). The Free Radical Spin-trap α-PBN Attenuates Periinfarct Depolarizations Following Permanent Middle Cerebral Artery Occlusion in Rats without Reducing Infarct Volume. Brain Res..

[86] Goda W., Satoh K., Nakashima M., Hara A., Niwa M. (2012). PBN Fails to Suppress in Delayed Neuronal Death of Hippocampal CA1 Injury Following Transient Forebrain Ischemia in Gerbils. Neurosci. Lett..

[87] Samadi A., Soriano E., Revuelta J., Valderas C., Chioua M., Garrido I., Bartolomè B., Tomassolli I., Ismaili L., Gonzàlez-Lafuente L. (2011). Synthesis, Structure, Theoretical and Experimental in vitro Antioxidant/Pharmacological Properties of α-Aryl, *N*-Alkyl Nitrones, as Potential Agents for the Treatment of Cerebral Ischemia. Bioorg. Med. Chem..

[88] Chioua M., Sucunza D., Soriano E., Hadjipavlou-Litina D., Alcàzar A., Ayuso I., Oset-Gasque M.J., Gonzàlez M.P., Monjas L., Rodrìguez-Franco M.I. (2012). α-Aryl-*N*-alkyl nitrones, as Potential Agents for Stroke Treatment: Synthesis, Theoretical Calculations, Antioxidant, Anti-inflammatory, Neuroprotective, and Brain-blood Barrier Permeability Properties. J. Med. Chem..

[89] Arce C., Díaz-Castroverde S., Canales M.J., Marco-Contelles J., Samadi A., Oset-Gasque M.J., González M.P. (2012). Drugs for Stroke: Action of Nitrone (Z)-*N*-(2-Bromo-5-hydroxy-4-methoxybenzylidene)-2-methylpropan-2-amine Oxide on Rat Cortical Neurons in Culture Subjected to Oxygen-glucose-deprivation. Eur. J. Med. Chem..

[90] Jiménez-Almarza A., Diez-Iriepa D., Chioua M., Chamorro B., Iriepa I., Martínez-Murillo R., Hadjipavlou-Litina D., Oset-Gasque M.J., Marco-Contelles J. (2019). Synthesis, Neuroprotective and Antioxidant Capacity of PBN-related Indanonitrones. Bioorg. Chem..

[91] Chamorro B., Diez-Iriepa D., Merás-Sáiz B., Chioua M., García-Vieira D., Iriepa I., Hadjipavlou-Litina D., López-Muñoz F., Martínez-Murillo R., González-Nieto D. (2020). Synthesis, Antioxidant Properties and Neuroprotection of α-Phenyl-*tert*-butylnitrone Derived Homobisnitrones in in vitro and in vivo Ischemia models. Sci. Rep..

[92] Diez-Iriepa D., Iriepa I., López-Muñoz F., Marco-Contelles J., Hadjipavlou-Litina D. (2022). Synthesis and Antioxidant Properties of Heterobisnitrones Derived from Benzene Dicarbaldehydes. Antioxidants.

[93] Diez-Iriepa D., Chamorro B., Talaván M., Chioua M., Iriepa I., Hadjipavlou-Litina D., López-Muñoz F., Marco-Contelles J., Oset-Gasque M.J. (2020). Homo-tris-nitrones Derived from α-Phenyl-*N*-tertbutylnitrone: Synthesis, Neuroprotection and Antioxidant Properties. Int. J. Mol. Sci..

[94] Chamorro B., Garcia-Vieira D., Diez-Iriepa D., Garagarza E., Chioua M., Hadjipavlou-Litina D., López-Muñoz F., Marco-Contelles J., Oset-Gasque M.J. (2021). Synthesis, Neuroprotection, and Antioxidant Activity of 1,1′-Biphenylnitrones as α-Phenyl-*N-tert*-butylnitroneanalogues in in vitro Ischemia Models. Molecules.

[95] Diez-Iriepa D., Iriepa I., Knez D., Gobec S., de los Ríos C., Bravo I., López-Muñoz F., Marco-Contelles J., Hadjipavlou-Litina D. (2022). Polyfunctionalized α-Phenyl-*tert*-butyl(benzyl)nitrones: Multifunctional Antioxidants for Stroke Treatment. Antioxidants.

[96] Escobar-Peso A., Martínez-Alonso E., Hadjipavlou-Litina D., Alcázar A., Marco-Contelles J. (2024). Synthesis, Antioxidant and Neuroprotective Analysis of Diversely Functionalized α-Aryl-*N*-alkyl Nitrones, as Potential Agents for Ischemic Stroke Therapy. Eur. J. Med. Chem..

